# Transformation of metal-organic frameworks for molecular sieving membranes

**DOI:** 10.1038/ncomms11315

**Published:** 2016-04-19

**Authors:** Wanbin Li, Yufan Zhang, Congyang Zhang, Qin Meng, Zehai Xu, Pengcheng Su, Qingbiao Li, Chong Shen, Zheng Fan, Lei Qin, Guoliang Zhang

**Affiliations:** 1 Institute of Oceanic and Environmental Chemical Engineering, State Key Lab Breeding Base of Green Chemical Synthesis Technology and Collaborative Innovation Center of Membrane Separation and Water Treatment of Zhejiang Province, Zhejiang University of Technology, Chaowang Road 18#, Hangzhou 310014, China; 2 Department of Materials Science and Engineering, College of Engineering, University of California, Berkeley, California 94720, USA; 3 Department of Chemical and Biological Engineering, College of Chemical and Biological Engineering, State Key Laboratory of Chemical Engineering, Zhejiang University, Hangzhou 310027, China; 4 Department of Chemical and Biochemical Engineering, College of Chemistry and Chemical Engineering, National Laboratory for Green Chemical Productions of Alcohols, Ethers and Esters, Key Lab for Chemical Biology of Fujian Province, Xiamen University, Xiamen 361005, China

## Abstract

The development of simple, versatile strategies for the synthesis of metal-organic framework (MOF)-derived membranes are of increasing scientific interest, but challenges exist in understanding suitable fabrication mechanisms. Here we report a route for the complete transformation of a series of MOF membranes and particles, based on multivalent cation substitution. Through our approach, the effective pore size can be reduced through the immobilization of metal salt residues in the cavities, and appropriate MOF crystal facets can be exposed, to achieve competitive molecular sieving capabilities. The method can also be used more generally for the synthesis of a variety of MOF membranes and particles. Importantly, we design and synthesize promising MOF membranes candidates that are hard to achieve through conventional methods. For example, our CuBTC/MIL-100 membrane exhibits 89, 171, 241 and 336 times higher H_2_ permeance than that of CO_2_, O_2_, N_2_ and CH_4_, respectively.

## Abstract (web summary)

Metal-organic frameworks (MOFs) are attracting increasing attention as membrane components for molecular sieving due to the range of desirable properties they exhibit. Here, the authors employ in situ cation substitution to transform MOF topologies, and endow the membranes with improved separation capabilities.

## Graphical abstract

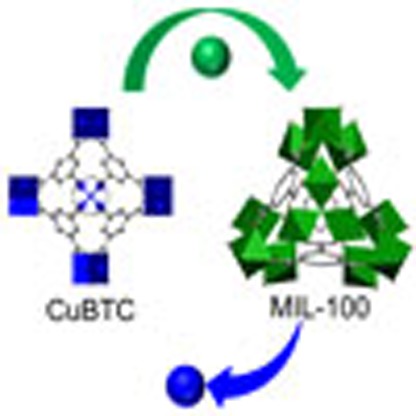

Molecular sieving membranes composed of silica^1^, zeolites^2^,^3^, graphene^4^,^5^ or metal-organic frameworks (MOFs)^6^,^7^ have intrinsic advantages, including both larger permeability and better selectivity, compared with those of conventional polymeric membranes^8^. MOFs consisting of metal ions or metal clusters coordinated with organic linkers have great potential as molecular sieves because of their large surface area, diverse structures and tailorable pore sizes. Until now, tens of thousands of MOFs have been reported and investigated^9^. However, compared with the total number of MOFs reports, those that can be assembled into continuous membranes are limited because of the necessary complex fabrication and activation procedures^10^. Substitution reaction chemistry has been extensively employed in various MOF modification procedures, notably to expand pore aperture or enhance adsorption of MOFs, by linker exchange or transmetalation^11^,^12^,^13^,^14^,^15^,^16^,^17^. However, MOFs synthesized through substitution protocols usually possess the same topological structure as their precursor, and no such investigation has focused on MOF membranes. Therefore, if we can establish a synthetic connection between different series of MOFs, we may greatly increase their potential in filtration applications.

Here we report a methodology for realizing the connection and complete transformation of different series of MOF membranes and particles based on multivalent cation substitution. This strategy combines three key concepts: (i) facile transformation of unstable and easily fabricated MOF particles to obtain the stable MOFs with completely different topology structure, which are usually fabricated in a relatively harsh synthetic conditions; (ii) *in situ* transformation of one common MOF membrane to another MOF membrane, which is hard to be synthesized in conventional methods at present; (iii) reducing the pore size through immobilizing the metal salt residue in cavities and exposing the appropriate crystal facets of the MOFs to achieve competitive molecular sieving ability by the transformation of MOF membranes. Our strategy can be used more generally to various MOF membranes and particles, but we exhibit our key findings here with two examples, one is the transformation of CuBTC to MIL-100, which takes the advantages of easy preparation and material stability^18^,^19^,^20^. Another is the transformation of CuBTC membrane to CuBTC/MIL-100 membrane, which provides a facile route to design promising candidates of MOF membrane for molecular sieving.

## Results

### Transformation of CuBTC to MIL-100

**Figure 1:**
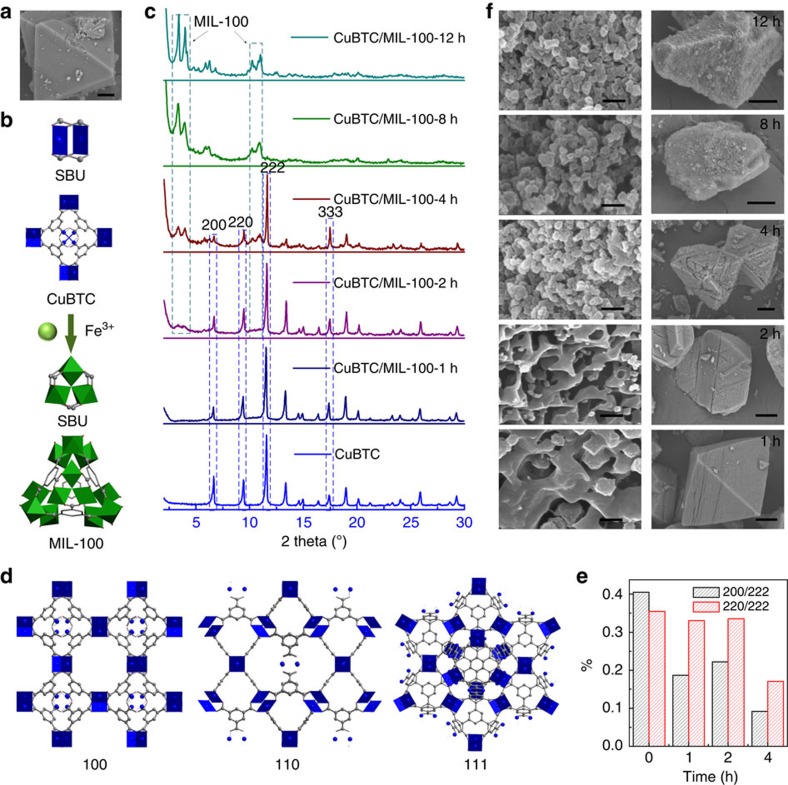
Transformation of CuBTC to MIL-100. (**a**) SEM image of as-synthesized octahedron CuBTC crystal. Scale bar, 10 μm. (**b**) Transformation procedure in metal centre and second building unit of CuBTC and MIL-100. The Cu and Fe coordination polyhedra are represented in blue and green, respectively, whereas the BTC links are depicted by sticks. (**c**) XRD patterns of CuBTC/MIL-100 with different transformation time. (**d**) CuBTC structure viewed at different facet. (**e**) Peak intensity ratios of {200}/{222} and {220}/{222} with the transformation time increasing. (**f**) SEM images of CuBTC/MIL-100 with different transformation time. The octahedron CuBTC crystal (bottom, 1 h) transformed into nanoparticle MIL-100 crystal (top,12 h). Scale bar, 200 nm (left); 20 μm (right).

We first conducted the transformation of CuBTC to MIL-100. CuBTC (also known as HKUST-1), a MOF with a cubic Pt_3_O_4_-type network, is composed of paddle wheel dimeric copper carboxylate units bridged by three-connecting 1,3,5-benzenetricarboxylate (BTC) linkers^18^. As one of the earliest reported and most studied MOF materials, it has been produced in large scale and used to fabricate continuous membranes^10^,^19^ despite its relatively poor chemical stability^20^. The MIL series of MOFs possess the excellent chemical stability, yet their synthetic conditions are much more rigorous compared with those of CuBTC^21^. For example, MIL-100(Fe) (Supplementary Fig. 1), composed of BTC and Fe-based centre with zeotype architecture^22^, is typically synthesized at 150 °C for 6 days and the precursor solution contains hazardous hydrofluoric acid, which is harmful to the environment. Although the preparation process of MIL-100(Fe) has been simplified considerably by reflux and microwave-assisted method at about 100 °C recently^23^,^24^ compared with CuBTC, which can be straightforwardly fabricated at low temperature^19^,^25^,^26^,^27^, the synthetic conditions and experimental equipment are still more rigorous. Moreover, new synthetic methods are desired highly to formation of MOF at room temperature^27^. Thus, we selected CuBTC and MIL-100 as main precursor and product to demonstrate the transformation between the MOFs. Scanning electron microscopy (SEM) image of CuBTC particle and transformation procedure in metal centre and second building unit are shown in Fig. 1a,b. The transformation was implemented by immersing CuBTC into FeCl_3_·6H_2_O solution at room temperature. All water, methanol, ethanol and *N*,*N*-dimethylformamide (DMF) were employed as the solvents. From the comparison between the simulated X-ray diffraction (XRD) of MIL-100 and XRD patterns of the prepared materials, we found that only the pattern of MIL-100 transformed in methanol showed all characteristic peaks similar to simulated XRD patterns, which demonstrated that the methanol was a relatively better solvent for achieving pure MIL-100 crystals with excellent crystallinity, but the three other solvents were not the same case (Supplementary Fig. 2). This phenomenon should be attributed to the balance among the diffusion rate of the solvent and Fe^3+^ ions in the pores of crystals, hydrolysis rate of CuBTC and substitution rate. CuBTC is unstable in water, and the Lewis acidic species of Fe^3+^ further accelerate the hydrolysis. Therefore, CuBTC was hydrolysed before the reaction was completed in aqueous solution. Compared with methanol, ethanol and DMF possess larger diameter and smaller polarity, which may conduce to much lower diffusion rate of reagents in CuBTC and substitution rate, and led to little cation substitution. Some monovalent and divalent cations were also employed to transform CuBTC, the result revealed that the MOF crystal structure had not been changed (Supplementary Fig. 3). Moreover, similar molar quantity of Cu^2+^ and BTC as CuBTC cannot be used to synthesize MIL-100 (Supplementary Fig. 4), demonstrating that the importance of the CuBTC framework and the transformation process was based on multivalent cation substitution.

### Transformation mechanism

**Figure 2:**
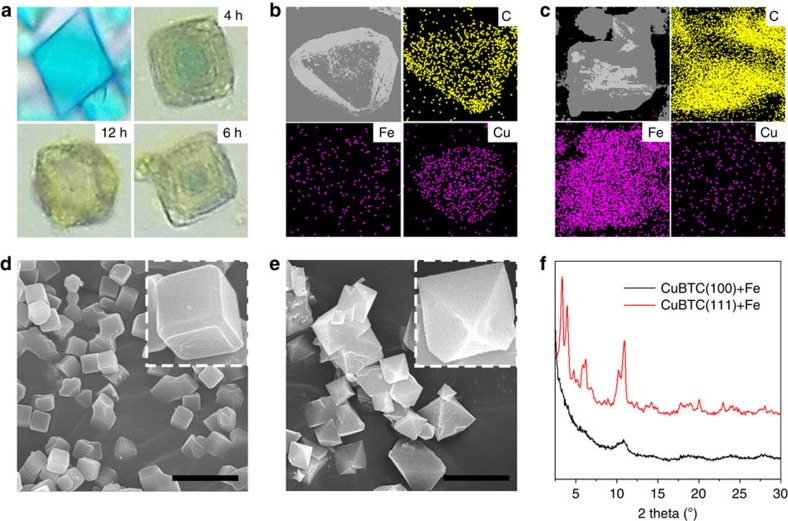
Transformation process from CuBTC to MIL-100. (**a**) Optical microscopy images of CuBTC with different transformation time, the transformation was carried out under standing. (**b**) EDS mapping of the CuBTC/MIL-100 after transformation for 4 h and purification in methanol, Fe/Cu ratio is 23.5%. (**c**) EDS mapping of the CuBTC/MIL-100 after transformation for 12 h and purification in methanol and hot water, Cu/Fe ratio is 0.4%. (**d**) SEM images of the cubic CuBTC with mainly exposed {100} facet. Scale bar, 5 μm. (**e**) SEM images of the small octahedron CuBTC with mainly exposed {111} facet. (**f**) XRD patterns of cubic CuBTC and small octahedron CuBTC after transformation. Scale bar, 5 μm.

We characterized the CuBTC/MIL-100 particles with different substitution times to investigate the reaction processes of transformation. After substitution for 1 h, the number of peaks in XRD pattern showed no big change (Fig. 1c). When the duration time was extended to 21 h, peaks for MIL-100 emerged. With the passage of time, the peak intensities of {200} and {220} degraded rapidly, and the peak intensity ratios of {200}/{222} and {220}/{222} also decreased dramatically (Fig. 1e). Moreover, it should be noted that the peak intensity of {333} also displayed a much lower degradation rate compared with other peaks. CuBTC exhibits three window structures, largest pores with diameter of 9 Å, moderate side pores with the diameter of 5 Å and smallest triangular shaped windows with the diameter of 3.5 Å (ref. 28). Correspondingly, the window size of {111} facets is 3.5 Å, which is smaller than the pore size of 5 and 9 Å in {100} facets (Fig. 1d). The octahedron crystal morphology and the much higher peak intensity of {222} compared with {200} and {220} reveal that the CuBTC has mainly exposed {111} facet^29^. Thus, by combining above experimental results and CuBTC structures, we can deduce the following reaction processes: when CuBTC crystals were immersed into the substitution solutions, the exposed {111} facets would greatly hinder the diffusion of the solvent and Fe^3+^ ions, this may be the reason for the little cation substitution of CuBTC in above-mentioned three solvents. After the copper ions were replaced by the Fe^3+^ ions, some MIL-100 nanocrystals formed on the surface of the CuBTC crystals, and subsequently the channel for diffusion of the solvent and Fe^3+^ ion was produced due to the larger window of MIL-100 and the gap between the two kinds of MOFs. As a result, the solvent and the Fe^3+^ ion would pass through the {100} facets quickly and replace the adjacent Cu^2+^ ions in CuBTC crystals to form MIL-100. Although the window size of {110} facets was the same as that of {111} facets, the {111} facets possessed the large atomic density and accordion-like channel, which greatly decreased the substitution rate and increased the diffusion resistance. This also led to the decrease of the value of {220}/{222}. The morphologies of these CuBTC/MIL-100 crystals are consistent with the XRD results and the deduction (Fig. 1f and Supplementary Figs 5 and 6). After substitution for 1 h, the crystals displayed that CuBTC/MIL-100 had superstructural composite surfaces and three-dimensional CuBTC shelf with interior MIL-100 nanocrystals. When the substitution time increased, the superstructure extended to the middle of the CuBTC crystals. After reaction for 81 h, all CuBTC were transformed into MIL-100. For identification of the inside structure of CuBTC/MIL-100 in transformation, the optical microscopy and energy dispersion spectroscopy (EDS) mapping images of CuBTC/MIL-100 with different transformation time were collected and presented in Fig. 2a–c. We can find that, with the extension of transformation times, the blue part of CuBTC became small but the shape was still octahedron. The optical microscopy images showed the lamellar structure, which was parallel to the boundary of CuBTC crystal, this may be attributed to the untransformed CuBTC with preferred {111} crystal facet, which was consistent with the results of XRD and showed the important role of {111} facet in transformation. After transformation for 12 h, the whole blue CuBTC was changed to yellow MIL-100. EDS mapping showed the similar phenomenon. When transformation time was extended to 12 h, the Cu/Fe ratio was reduced to 0.4%. These results also revealed that the transformation was conducted from outside to inside. To further demonstrate the importance of the exposed {111} facet for transformation, we synthesized the cubic CuBTC with mainly exposed {100} facet as previous study (Fig. 2d)^30^, and used it to obtain the MIL-100. The XRD pattern revealed that this transformation failed to obtain the MIL-100 (Fig. 2f). Compared with the above successful transformation, two differences may lead to the unsuccessful transformation, the difference in exposed facet and the difference in CuBTC particle size. We further prepared the small octahedron CuBTC by simply stirring the precursor at room temperature (Fig. 2e)^26^, and found that this material could be transformed to be MIL-100 successfully. This results verified that the difference in exposed facet was the main reason of the failing transformation of the cubic CuBTC with mainly exposed {100} facet, rather than the difference in CuBTC particle size. The experiment also showed that the MIL-100 can be prepared by transformation of the small octahedron CuBTC in mild route at room temperature without using any special equipment (Supplementary Table 1).

### Universality of transformation

To present the versatility of this strategy, we transformed the MOF-5 to CuBDC by Cu^2+^ substitution and transformed the CuBDC to MIL-53 by Fe^3+^ substitution. In this process, MOF-5 was a face-centred cubic material, formed by Zn-based metal cluster and 1,4-ben-zenedicarboxylate (BDC) linker^31^, and can be synthesized at room temperature by adding triethylamine^32^; CuBDC consisted of paddle wheel dimeric copper carboxylate units interconnected by BDC^33^, and was usually fabricated in DMF at 110 °C or in acetonitrile system^33^,^34^,^35^,^36^; MIL-53 was formed by FeO_4_(OH)_2_ clusters with BDC^37^, and prepared typically in DMF or in water at high temparature^38^,^39^,^40^. The crystalline structures of these MOFs are presented in Supplementary Fig. 7. The XRD patterns and typical SEM images reveal the successful transformation (Supplementary Figs 7, 8 and 9). Because of the mild and environment-friendly process, the complex synthesis condition of some MOFs can be simplified greatly by transformation, which is of benefit to the scalable production of these MOFs.

### Property of transformed MIL-100

**Figure 3:**
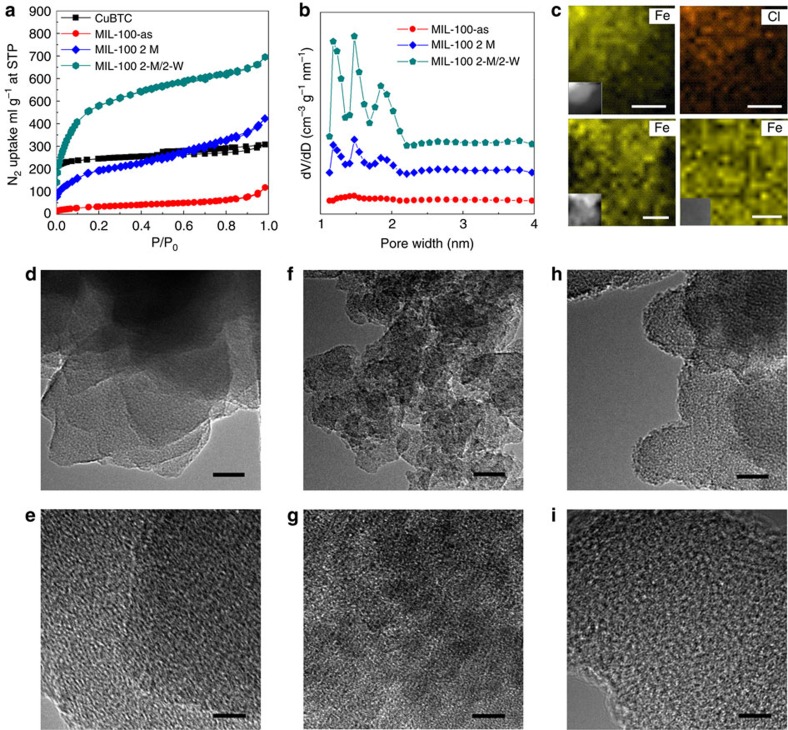
Characterization of MIL-100. (**a**) N_2_ adsorption–desorption isotherms on MIL-100 with various purification cycles, showing the specific surface area reduced with the purification cycle, two cycles in methanol (2-M), two cycles in methanol and then in hot water at 70 °C (2-M/2-W). (**b**) Corresponding pore size distributions calculated by DFT method. (**c**) EDS mapping of the MIL-100 with different post-processing. Top two images: as-synthesized MIL-100 and demonstrating the well-dispersed chlorine, scale bar, 20 nm; bottom left image: as-synthesized MIL-100 after calcination at 200 °C for 4 h, scale bar, 10 nm; bottom right image: MIL-100 after purification; scale bar, 5 nm. (**d**,**e**) TEM images of as-synthesized MIL-100, showing the amorphous FeCl_3_ in the cavities. (**f**,**g**) TEM images of as-synthesized MIL-100 after calcination at 200 °C for 4 h, indicating the presence of nanoclusters in the cavities of the MIL-100. (**h**,**i**) TEM images of MIL-100 after purification, indicating clear porous structure and pure framework. Scale bar, 20 nm (in **d**,**f**,**h**); 5 nm (in **e**,**g**,**i**).

We further studied the features of the transformed MIL-100. Nitrogen adsorption experiments reveal that the CuBTC sample has Brunauer–Emmett–Teller (BET)-specific surface area of 960 m^2^ g^−1^ and pore volume of 0.48 cm^3^ g^−1^ (Fig. 3a). After transformation, BET-specific surface area and pore volume increase obviously to 1880, m^2^ g^−1^ and 1.07 cm^3^ g^−1^, respectively, and the hysteresis loop reveals the existence of mesopores in MIL-100 (ref. 41). These values are consistent with those reported in previous studies (Supplementary Table 1), and demonstrate the good porosity of the prepared MIL-100. The DFT pore size distribution curve displays that the prepared MIL-100 possesses the multi-scale pore structure (Fig. 3b), as similar results have been observed in many previous studies^24^,^42^,^43^,^44^. The small skewing in O 1s X-ray photoelectron spectroscopy demonstrates the successful transformation of the C–O–Cu bond in CuBTC to C–O–Fe bond in MIL-100 (Supplementary Fig. 10). Thermal analysis shows the same good thermal stability of the transformed MIL-100 and normal thermal degradation curve as those in previous reports^22^ (Supplementary Fig. 11). From the SEM images (Fig. 1f), we can find that MIL-100 nanocrystals have a relatively small size in the range of 30–60 nm. The integrated MIL-100 particle after treated by ultrasonic treatment demonstrates that MIL-100 nanoparticles grew together tightly, rather than simple accumulation (Supplementary Fig. 12). These features exhibit that MIL-100 was hierarchically porous material. Moreover, we found that some Fe-based materials can be kept in cavities by insufficient purification to reduce the pore size of the obtained MOFs. With the decrease of purification cycle, the adsorption–desorption isotherms change from type-I/IV to type-II. The specific surface area and pore volume reduce to 122 m^2^ g^−1^ and 0.18 cm^3^ g^−1^ (Fig. 3a), respectively, and the pore size also decreases obviously (Fig. 3b). The relative intensities of XRD patterns of the as-synthesized MIL-100 also show a strong reduction compared with the MIL-100 after purification, especially at low angles (Supplementary Fig. 13), which may be attributed to the impregnation of amorphous FeCl_3_ (refs 21, 42). Combining with the vast existence of well-dispersed chlorine (10.2%) in the as-synthesized MIL-100 (Supplementary Table 2 and Fig. 3c), and the small amount of chlorine (0.2%) in MIL-100 after purification in methanol and hot water, we come to a conclusion that the cavities of as-synthesized MIL-100 have been occupied by highly dispersed amorphous FeCl_3_, which can be utilized to artificially control the pores of the transformed MOFs. To further prove the existence of FeCl_3_ in the cavities of as-synthesized MIL-100, it was calcined at 200 °C for 41 h. The chlorine displays a great reduction (74.9%) and the XRD patterns show some new peaks of Fe_2_O_3_ for as-synthesized MIL-100 (Supplementary Fig. 14). All these reveal that the amorphous FeCl_3_ in the cavities has been converted to Fe_2_O_3_ nanoclusters. To further identify the formation of FeCl_3_ and the structure of the MIL-100, we collected the transmission electron microscopy (TEM) images of as-synthesized MIL-100, as-synthesized MIL-100 after calcination at 200 °C for 4 h and MIL-100 after purification in methanol and hot water (Fig. 3d–i). TEM images clearly indicate that abundant of amorphous materials are left in the cavities of as-synthesized MIL-100 (Fig. 3d,e), however, the purified MIL-100 has clear porous structure and pure framework (Fig. 3h,i), there are almost no residues left in the cavities of purified MIL-100. For as-synthesized MIL-100 after calcination, the uniformly dispersed dark spots in TEM images demonstrates that the Fe_2_O_3_ nanoclusters are immobilized inside the cavities of insufficiently purified MIL-100 (Fig. 3f,g). The nanoclusters in cavities may be beneficial to improve the activity of MOFs. In addition, the reduced pore size will be very important for the performance of molecular sieve.

### Transformation of MOF membrane

**Figure 4:**
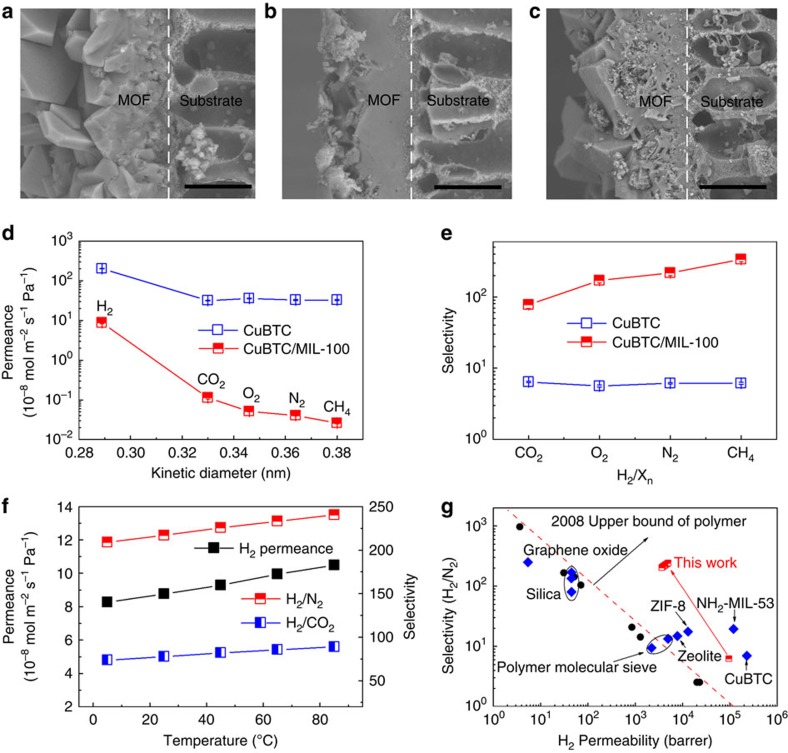
Transformation of CuBTC membrane and their performance. (**a**–**c**) SEM images of original CuBTC membrane, transformed CuBTC/MIL-100 membrane and transformed CuBTC/MIL-100 membrane after purification, respectively. Scale bar, 20 μm. (**d**,**e**) Gas permeance and selectivities of the CuBTC and CuBTC/MIL-100 membranes. All the average permeation results with standard deviation were calculated from three measurement data. (**f**) Effect of temperature on H_2_ permeance and H_2_/CO_2_ and H_2_/N_2_ selectivities for CuBTC/MIL-100 membrane. (**g**) Comparison of CuBTC/MIL-100 membrane with polymeric^8^,^47^, silica^1^, zeolite^3^, other MOF^46^,^48^,^49^ and graphene oxide membranes^5^ for H_2_/N_2_ system. 1 barrer=3.348 × 10^*−*16^ mol m^*−*2^ s^*−*1^ Pa^*−*1^, the red dotted line is the Robeson's upper-bound reported in 2008 (ref. 8).

After demonstrating the transformation of MOFs, our strategy was further employed to transform MOF membranes. CuBTC membrane was synthesized on polymeric hollow fibre by solvothermal method and used as precursor^45^ (Supplementary Fig. 15). Figure 4a–c presents the SEM images of CuBTC membrane, as-synthesized CuBTC/MIL-100 membrane and CuBTC/MIL-100 membrane after purification. The CuBTC layer with thickness of ∼20 μm is anchored to the substrate continuously. The octahedron morphology of crystal demonstrates that the CuBTC membrane also has mainly exposed {111} facet. After transformation, the as-synthesized CuBTC/MIL-100 membrane seems to become denser due to the FeCl_3_ residue (Supplementary Figs 16 and 17). However, after the FeCl_3_ was removed, the massive MIL-100 nanoparticles accompanied by CuBTC flake occurred on the cross-section of purified CuBTC/MIL-100 membrane (Fig. 4c and Supplementary Fig. 16). EDS mapping demonstrates the incomplete transformation (Supplementary Fig. 17). The existence of the peak with 2 theta at 11.6^o^ in XRD pattern also shows the incomplete transformation and the residual CuBTC with exposed {111} facet (Supplementary Fig. 18). This is consistent with the phenomenon observed in incompletely transformed MOF particles, and can be explained by the smaller mass transfer-specific surface area exposed to the solution of membrane. When the duration time was extend to 48 h, all the CuBTC were transformed to MIL-100 (Supplementary Fig. 16).

### Performance of transformed membrane

To investigate the performance of the prepared MOF membranes by transformation, we used the constant-pressure method to measure H_2_ (kinetic diameter: 0.289 nm), CO_2_ (0.33 nm), O_2_ (0.346 nm), N_2_ (0.364 nm) and CH_4_ (0.38 nm) permeances through the two dense membranes^4^. The CuBTC membrane exhibited H_2_ permeance and selectivities of H_2_/X_n_ (X_n_: other gases) in the range of 5.5–6.5, similar to those previously reported^46^ (Fig. 4d,e). For the transformed CuBTC/MIL-100 membrane, the permeances of all gases were smaller than those of CuBTC membrane, and the largest H_2_ permeance was 8.8 × 10^−8^ mol m^−2^ s^−1^ Pa^−1^. However, the selectivities of H_2_/CO_2_, H_2_/O_2_, H_2_/N_2_ and H_2_/CH_4_ displayed great improvement and reached about 77.6, 170.6, 217.0 and 335.7, respectively. Moreover, the selectivities increased with temperature and reached 89.0 and 240.5 for H_2_/CO_2_ and H_2_/N_2_ at 85 °C, respectively, as the H_2_ permeances grew faster than other gases. Meanwhile, the H_2_ permeance also increased to 10.5 × 10^−8^ mol m^−2^ s^−1^ Pa^−1^ (Fig. 4f). The transformed membrane also presented excellent durability, which maintained good performance with only small fluctuation over a 192-h period (Supplementary Fig. 19). Our transformed molecular sieving membrane had much better performance than polymeric membranes and can easily exceed the Robeson's upper-bound reported in 2008 for all H_2_/CO_2_, H_2_/N_2_ and H_2_/CH_4_ systems^8^,^47^. With regard to porous zeolites^3^ and conventional MOF membranes^46^,^48^,^49^, the transformed CuBTC/MIL-100 membrane showed superior performance in selectivities (Fig. 4g and Supplementary Fig. 20). Even compared with the silica, graphene oxide and Zn_2_(bim)_4_ nanosheet membranes reported, which exhibited great selectivities for H_2_/CO_2_ or/and H_2_/N_2_ systems^1^,^4^,^5^,^7^, our membrane also exhibited competitive performance. The transformed CuBTC/MIL-100 membrane possessed excellent H_2_ permeability as high as ∼4,000 barrer as well as good stability under high trans-membrane pressure of 0.25 MPa (Supplementary Fig. 21), while the reported membranes were usually operated under very low trans-membrane pressure of 0.1 or even 0 MPa to prevent possible cracks. In binary mixture separation, because of the competitive adsorption, the membrane showed a little smaller H_2_ permeance and selectivities compared with the values measured by constant-pressure method, but the membrane still showed the competitive selectivities (Supplementary Table 3). Moreover, the synthetic procedure has good reproducibility, which has been demonstrated by the similar separation performance of two further additional membranes. We speculate that the good molecular sieving performance of transformed CuBTC/MIL-100 membrane is caused by the following three factors (Supplementary Fig. 22): First, the transformed membrane is continuous, which is the basis for good performance. Second, as mentioned above, the reagents first enter into of the {100} facets of CuBTC and then the transformation occurs, the peak at 11.6^o^ in XRD pattern displays the exposed {111} facets of the residual CuBTC, so the triangular shaped window with a diameter of 3.5 Å in the residual and the exposed {111} facets is a main channel for gas to pass through. This window size is similar to the kinetic diameter of the CO_2_, O_2_ and N_2_, and is much larger and smaller than that of H_2_ and CH_4_, respectively. Thus, H_2_ molecules can penetrate the transformed membrane quickly. Third, the massive amorphous FeCl_3_ can fill the gaps between MIL-100 and CuBTC and also occupy the cavities and pores of MOFs. As a result, the void interfaces can be eliminated, the gas channel can be reduced and the gas selectivities of the transformed membrane are increased. Because of the tailorable pore sizes of MOF materials and the diversity in membrane synthesis, we envisage there would be some optimal MOFs and proper preparation conditions, where the membranes with smaller thickness and better separation performance can be achieved by transformation in the future.

## Discussion

We have developed a facile and general methodology for realizing the connection and complete transformation of different series of MOF membranes and particles based on multivalent cation substitution. Through this strategy, the unstable and easily fabricated MOF particles can be transformed to obtain the stable MOFs with completely different topology structure, which are usually fabricated in relatively harsh synthetic conditions. The common MOF membranes can also be *in situ* transformed to another MOF membrane, which is hard to be synthesized in conventional methods so far. The pore size can be controlled through immobilizing the metal salt residue in cavities and the appropriate crystal facets of the MOFs can be exposed to achieve competitive molecular sieving ability by the transformation. The typically transformed CuBTC/MIL-100 membrane with good stability exhibits 89, 171, 241 and 336 times higher H_2_ permeance than that of CO_2_, O_2_, N_2_ and CH_4_, respectively. The method can be used more generally to various MOF membranes and particles with great potential in wide applications.

## Methods

### Synthesis of CuBTC

Cu(NO_3_)_2_·3H_2_O and BTC were dissolved in water and ethanol, respectively. For crystallization, the two solutions were mixed and transferred into a Teflon-lined autoclave. Then, the reaction mixture was heated. After cooling to room temperature, the blue particles were isolated by centrifugation. Eventually, CuBTC particles were washed by ethanol and methanol and dried.

### Synthesis of MOF-5

Zn(NO_3_)_2_·6H_2_O and BDC were dissolved into DMF, respectively. For crystallization, the two solutions were mixed and heated in an autoclave^50^. After cooling to room temperature, the white particles were separated by centrifugation. Eventually, the prepared particles were washed by DMF and methanol and dried.

### Synthesis of CuBDC

Cu(NO_3_)_2_·3H_2_O and BDC were dissolved into the DMF, respectively. For crystallization, the two solutions were mixed and heated^34^. After cooling, the blue particles were isolated by centrifugation. Eventually, CuBTC particles were washed by DMF and methanol and dried.

### Synthesis of CuBTC with mainly exposed {100} facet

Cu(NO_3_)_2_·3H_2_O and lauric acid were dissolved into ethanol (10 ml), BTC was also dissolved in ethanol (10 ml). For crystallization, the two solutions were mixed and heated at 150 °C for 24 h (ref. 30). After natural cooling, the resulting blue powders were isolated by centrifugation and washed with ethanol.

### Synthesis of small CuBTC with mainly exposed {111} facet

Cu(NO_3_)_2_·3H_2_O and BTC were dissolved in water and ethanol, respectively, to obtain the solutions with concentration of 80 mmol l^−1^. For crystallization, the BTC solution was added into the metal salt solution and stirred at room temperature for 18 h. After reaction, the blue CuBTC crystals were separated by centrifugation and washed by ethanol.

### Transformation of CuBTC to MIL-100

FeCl_3_·6H_2_O (2.00 g) was dissolved in different solvents. CuBTC (0.1 g) was dispersed into the above solution. For transformation, the obtained suspension was fixed into a shaker. Reaction was carried out at room temperature. The reaction time was 1, 2, 4, 8 or 12 h. After reaction, the prepared particles were separated by centrifugation (5,000 r.p.m., 5 min) and purified with methanol. The obtained MIL-100 was purified with various purification cycles, two cycles in methanol (2-M), two cycles in methanol and then in hot water at 70 °C (2-M/2- W). To further identify the nanoclusters in MIL-100, the obtained particles were dried and calcined at 200 °C for 4 h. As comparison, water, ethanol and DMF were all employed as the solvent for transformation. To demonstrate the importance of the CuBTC and that the transformation was not a hydrolysis-recrystallization process, the Cu(NO_3_)_2_·3H_2_O (0.124 g) and BTC (0.066 g) were applied to displace the CuBTC, which possesed the same molar quantity as the CuBTC. The result revealed that the CuBTC was vital for transformation.

### Transformation of MOF-5 to CuBDC

MOF-5 (0.1 g) was dispersed into Cu(NO_3_)_2_·3H_2_O methanol solution (0.05 g ml^−1^). The above suspension was fixed into a shaker. Transformation was carried out at room temperature. The prepared blue particles were collected by centrifugation and purified by methanol and dried at 80 °C.

### Transformation of CuBDC to MIL-53

CuBDC (0.1 g) was dispersed into FeCl_3_·6H_2_O methanol solution (0.05 g ml^−1^). The above suspension was fixed into a shaker. Transformation was carried out at room temperature for 12 h. The prepared particles were collected by centrifugation and purified by methanol and dried at 80 °C.

### Synthesis of CuBTC membrane

For dopamine modification^48^, the polyvinylidene difluoride (PVDF) was washed using water and dried at room temperature to remove impurities on membrane surface, and levodopa was added in 10 mM Tris-HCl to obtained transparent solution. Then, the PVDF hollow fibre was immersed in the prepared solution for dopamine deposition. After deposition, the membrane was washed using water and dried at atmosphere. For non-activation ZnO array growth, 0.592 g of zinc nitrate hexahydrate, 0.135 g of sodium formate and 0.245 g of 2-methyl-imidazole were dissolved in methanol and transferred into an autoclave. The modified PVDF hollow fibre was immersed vertically into the mixture solution by using a self-made Teflon holder. Then, the autoclave was sealed and heated at 80 °C. After reaction for 12 h, PVDF hollow fibre with non-activation ZnO array was taken out and washed by ultrasound for 60 s to remove the loose powder. Ultimately, the hollow fibre was washed by methanol and dried at atmosphere. For synthesis, Cu(NO_3_)_2_·3H_2_O (0.5 g) and BTC (0.25 g) were dissolved in water and ethanol, respectively. These two solutions were mixed to obtain a clear precursor solution. The mixed solution was poured into an autoclave, and the PVDF hollow fibre with non-activation ZnO array was also placed vertically in the autoclave by using a self-made Teflon holder. The crystallization was executed at 85 °C for 48 h. After crystallization, the hollow fibre membrane was taken out, washed with ethanol and dried at room temperature.

### Transformation of CuBTC membrane

FeCl_3_·6H_2_O (1.00 g) was dissolved into the methanol. CuBTC hollow fibre membrane was soaked into the prepared solution at room temperature for 12 or 48 h. After reaction, the membrane was taken out and dried directly for obtaining the dense CuBTC/MIL-100 membrane. As comparison, the membrane after transformation was immersed in the pure methanol to remove the excess FeCl_3_ component.

### Gas permeation measurement

The gas permeation properties of the membrane were studied by constant-pressure, variable-volume method^4^. The dense membranes were put in a permeation module and sealed by epoxy glue. The effective area was calculated by the outer surface. The measured gas was used to rinse the permeation module. The feed gas and permeate gas were fed and collected at the shell side and tube side of the membrane, respectively. Upstream pressure and downstream pressure were 2 and 1 bar (atmosphere conditions), respectively, and transmembrane pressure was 1 bar. The experiment was carried out with different kinetic diameters in the following order: H_2_ (0.289 nm), CO_2_ (0.33 nm), O_2_ (0.346 nm), N_2_ (0.364 nm), CH_4_ (0.38 nm), CH_4_, N_2_, O_2_, CO_2_ and H_2_. Gas flow rates were measured by a bubble flow-metre. The gas permeation data were calculated by averaging the measured values of two cycles. The data were read and recorded until the system running stably. Gas permeance (*P*, mol m^−2^ s^−1^ Pa^−1^) and gas permeability (*P*_G_*, Barrer=*3.348 × 10^−16^ mol m^−2^ s^−1^ Pa^−1^) were calculated by using the following equations:




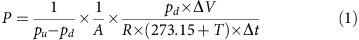







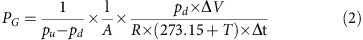




where *p*_u_ and *p*_d_ are the upstream pressure and downstream pressure, respectively, *A* and *l* are the membrane effective area and membrane thickness (thickness of the MOF layer), *R* and *T* are the gas constant value and temperature (Celsius), Δ*V* and Δ*t* are the volume of the gas through the membrane and the corresponding time. The permselectivity (*α*) is defined as the ratio of two kinds of gas permeances.




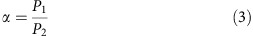




### Characterization

XRD patterns were recorded by a PANalytical X' Pert PRO X-ray diffractometer with Cu Kα radiation (*λ*=0.154056, nm) at 40 kV and 40 mA. A field-emission scanning electron microscope (S-4700, Hitachi) was used to observe the morphologies of the membranes. Accelerating voltage was 15 kV. The attached X-ray EDS (GENESIS4000, EDAX) was applied to analyse the element content of prepared MIL-100 particle after tabletting. To keep the cross-section morphology the MOF membranes, it was freeze-fractured in liquid nitrogen. All the prepared samples were coated with an ultrathin layer of platinum using an ion sputter coater to minimize charging effects. X-ray photoelectron spectroscopy experiments were performed on a RBD upgraded PHI-5000C ESCA system (Perkin Elmer) with an incident radiation of monochromatic Mg Kα X-rays (hν=1253.6 eV) at 250 W. The spectra of all the elements were collected by using RBD 147 interface (RBD Enterprises). A JEM-2100 (JEOL Co.) operated with accelerating voltage of 200 kV was used for obtaining the TEM images. TG measurements were executed on a thermal gravimetric analyser (PERKIN ELMER, Model TGA 7). The samples were heated from 25 or 40 to 700 °C with a heating rate of 20 °C min^−1^ under a flow of synthetic air with a flow rate of 20 ml min^−1^. N_2_ adsorption–desorption isotherms were measured on a Micromeritics-Accelerated Surface Area and Porosimetry system (ASAP 2020M+C, Micromeritics Instrument Co.). Measurements were carried out at 77 K held using a liquid nitrogen bath. The samples were degassed in vacuum at 150 °C for 12 h before the analysis. BET method was used to calculate the specific surface areas in the P/P_0_ range of 0.05–0.1. DFT was used to obtaining the pore-size distributions.

## Additional information

**How to cite this article:** Li, W. *et al*. Transformation of metal-organic frameworks for molecular sieving membranes. *Nat. Commun.* 7:11315 doi: 10.1038/ncomms11315 (2016).

## Supplementary Material

Supplementary Information Supplementary Figures 1-22, Supplementary Tables 1-3 and Supplementary References.
