# Supplementary material for: Transformation of metal-organic frameworks for molecular sieving membranes
Source: Nat Commun. 2016 Apr 19;7:11315. doi: 10.1038/ncomms11315 (PMC4838892; doi:10.1038/ncomms11315)
Supplement: Supplementary Information — Supplementary Figures 1-22, Supplementary Tables 1-3 and Supplementary References. [file ncomms11315-s1.pdf]

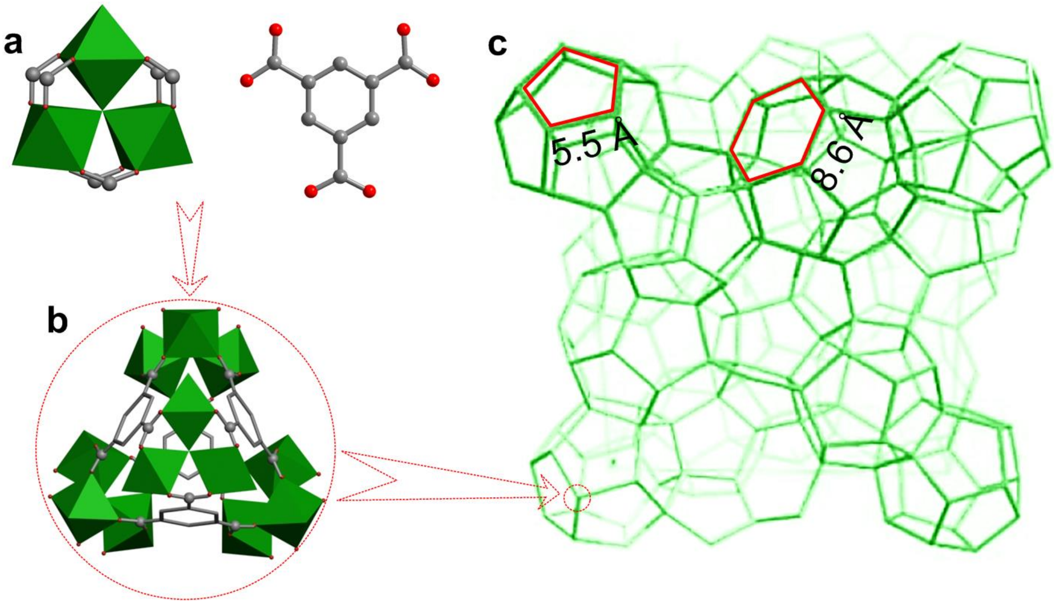

Supplementary Figure 1. Crystal structure of MIL-100. a, Trimer of Fe-based octahedra and 1,3,5-benzenetricarboxylate (Fe: green; C: black; O: red). **b**, Second building unit of MIL-100. **c**, Scheme of one unit cell of MIL-100.

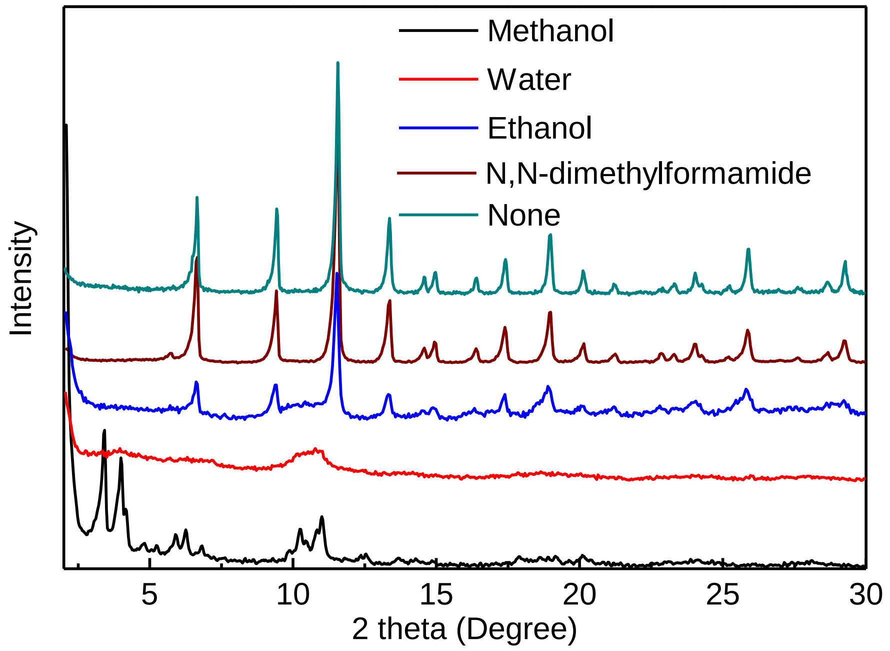

Supplementary Figure 2. XRD patterns of CuBTC/MIL-100 transformed in various solvent. XRD data demonstrate that the methanol is an excellent solvent for achieving pure MIL-100 crystals. The water hydrolyzed the CuBTC before the start of transformation. The CuBTC showed less transformation for ethanol as the solvent compared with methanol.

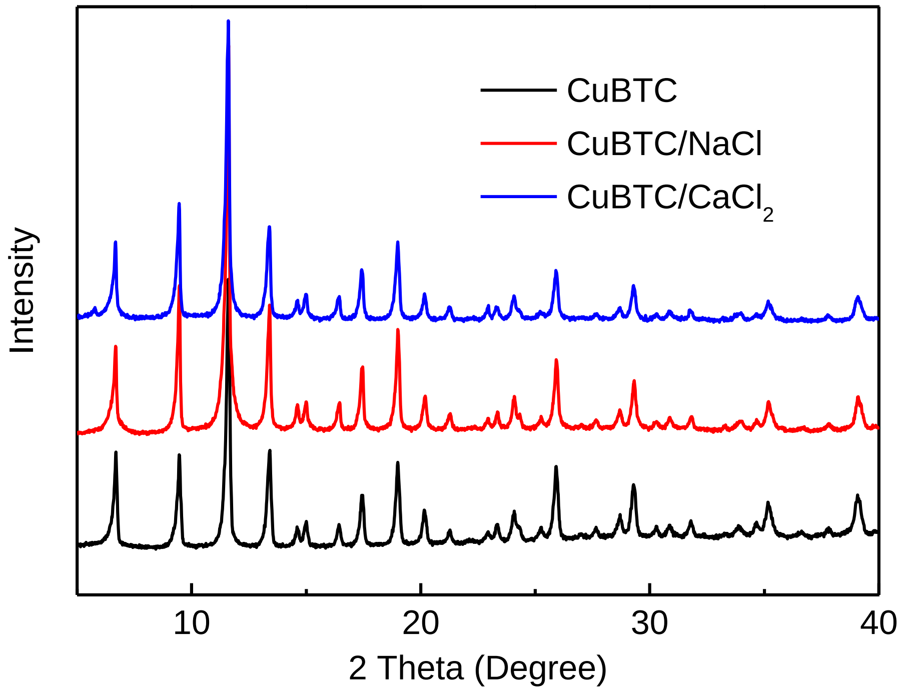

Supplementary Figure 3. XRD patterns of CuBTC transformed by Na^{+} and Ca^{2+} ions. Compared with the transformation by Fe^{3+}, this fabrication procedure had no change, but the $FeCl_3$ $6H_2O$ was replaced by NaCl and $CaCl_2$. The result reveals that these cations cannot change the crystal structure of the CuBTC, which is in agreement with the Irving-Williams series of the stability for discrete metal complexes[1].

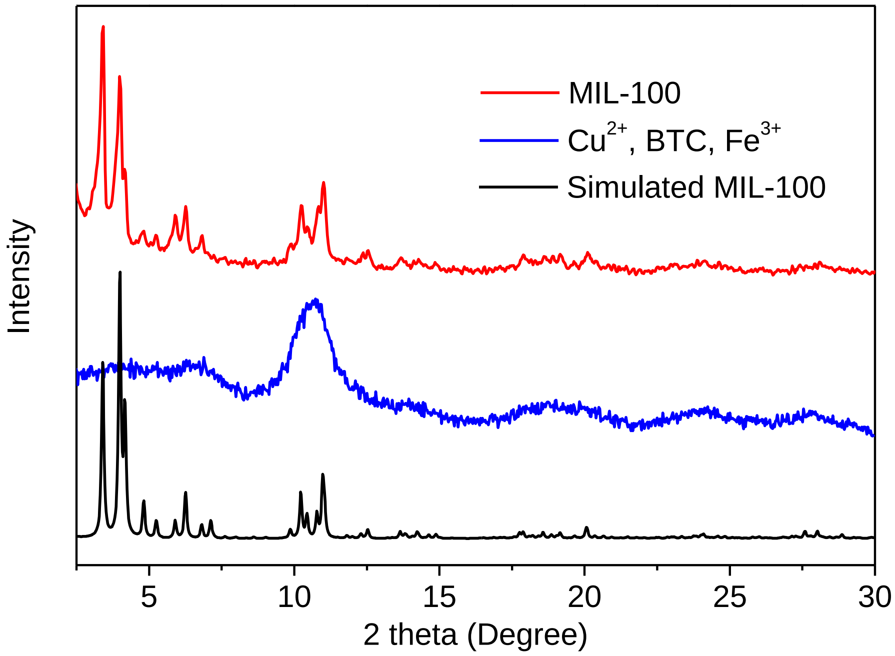

Supplementary Figure 4. XRD patterns of MIL-100 transformed by CuBTC, and Cu^{2+} and BTC. For the latter, the fabrication procedure was consistent to the former, but the CuBTC (0.1 g) were replaced by $Cu(NO_3)_2 \cdot 3H_2O$ (0.124) and BTC (0.066 g), which had the same molar quantity as CuBTC. The XRD pattern shows that the MIL-100 cannot be fabricated by the mixture of Cu^{2+}, BTC and Fe^{3+}. This experiment reveals the importance of the CuBTC, and demonstrates the transformation process is base on multivalent cation substitution.

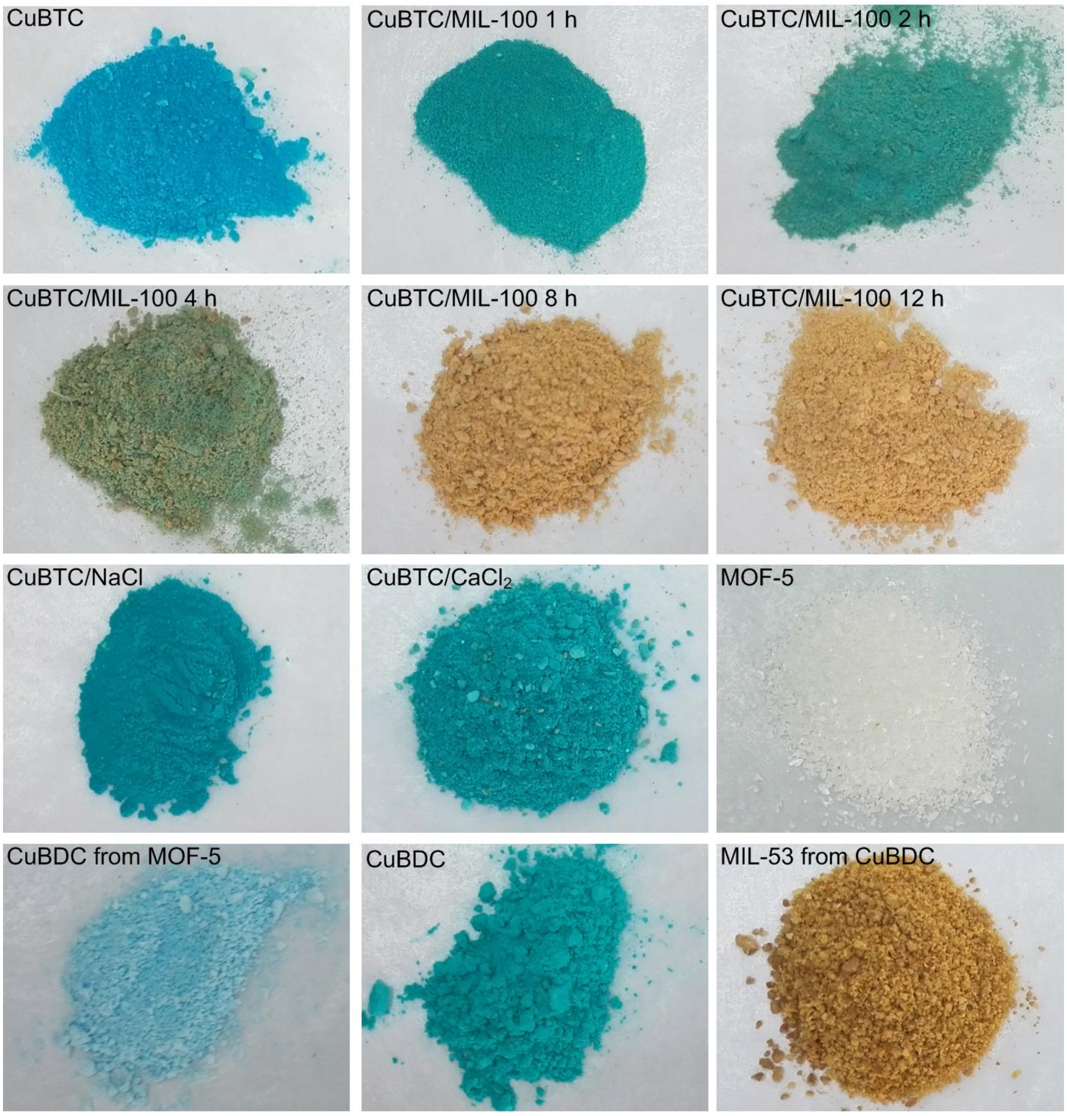

Supplementary Figure 5. Photographs of original MOF materials and MOF materials derived from transformation. From the left to right and top to down is CuBTC, CuBTC/MIL-100 transformed for 1 h, CuBTC/MIL-100 transformed for 2 h, CuBTC/MIL-100 transformed for 4 h, CuBTC/MIL-100 transformed for 8 h, MIL-100 transformed for 12 h, CuBTC/NaCl, CuBTC/$CaCl_2$, MOF-5, transformed CuBDC from MOF-5, synthesized CuBDC and transformed MIL-53 from CuBDC.

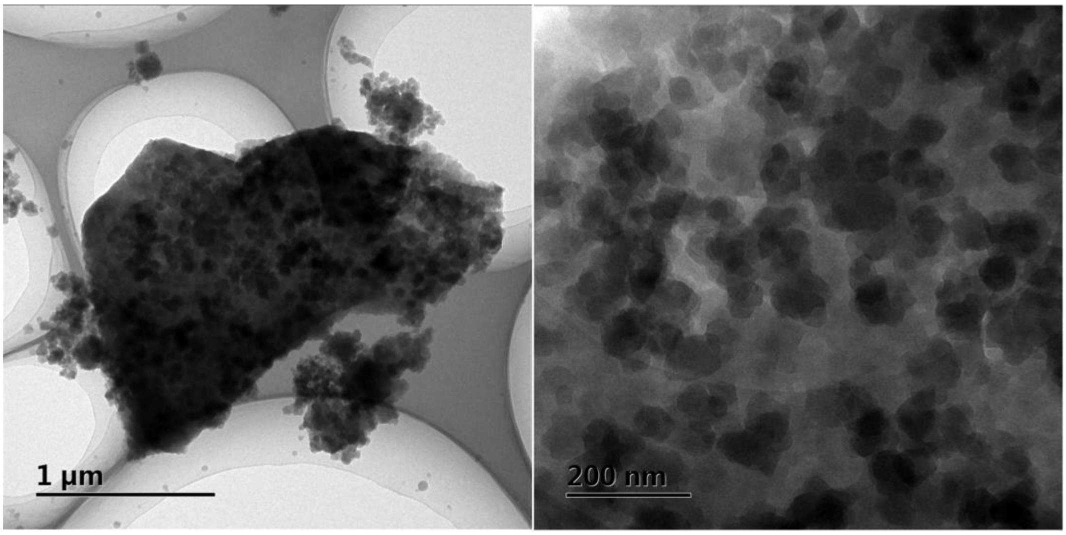

Supplementary Figure 6. TEM images of the CuBTC/MIL-100 with transformation time of 4 h. These images indicate that the dark MIL-100 nanocrystals existed in CuBTC particle.

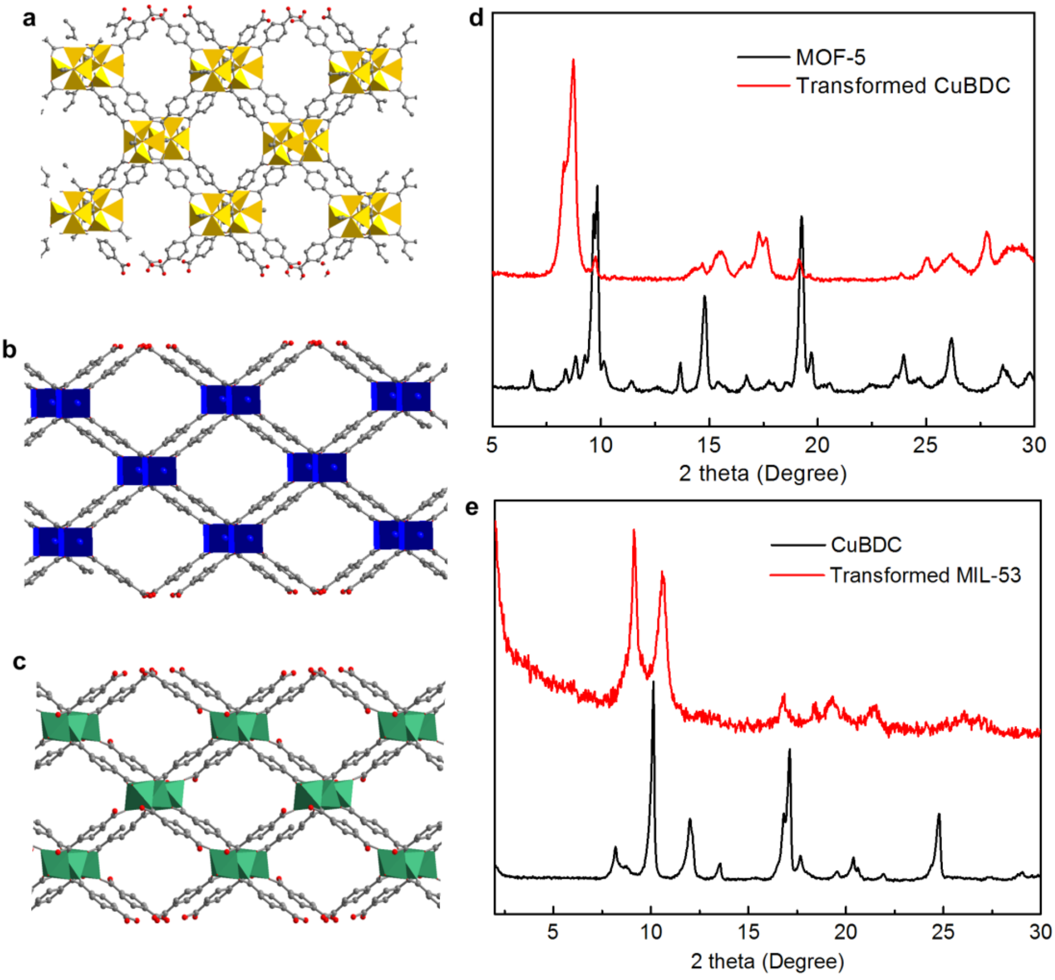

Supplementary Figure 7. Crystal structures of MOFs and XRD patterns of the original and transformed MOFs. a, MOF-5. **b**, CuBDC. **c**, MIL-53. **d**, XRD patterns of original MOF-5 and transformed CuBDC, CuBDC transformed by Cu^{2+} substitution from MOF-5. **e**, XRD patterns of original CuBDC and transformed MIL-53, MIL-53 transformed by Fe^{3+} substitution from CuBDC. The difference of original CuBDC and transformed CuBDC in XRD patterns should be attributed to the difference in synthetic solvents, DMF for original CuBTC and methanol for transformed CuBDC[2].

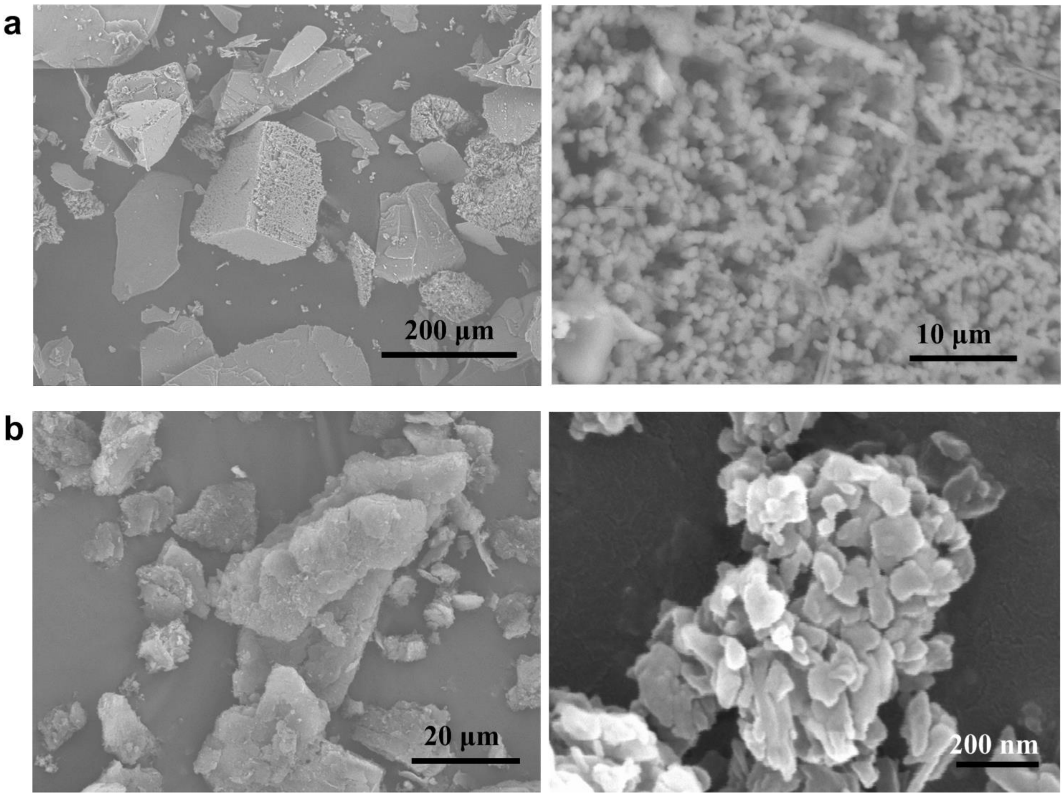

Supplementary Figure 8. SEM images of the original and transformed MOFs. a, MOF-5. **b**, Transformed CuBDC.

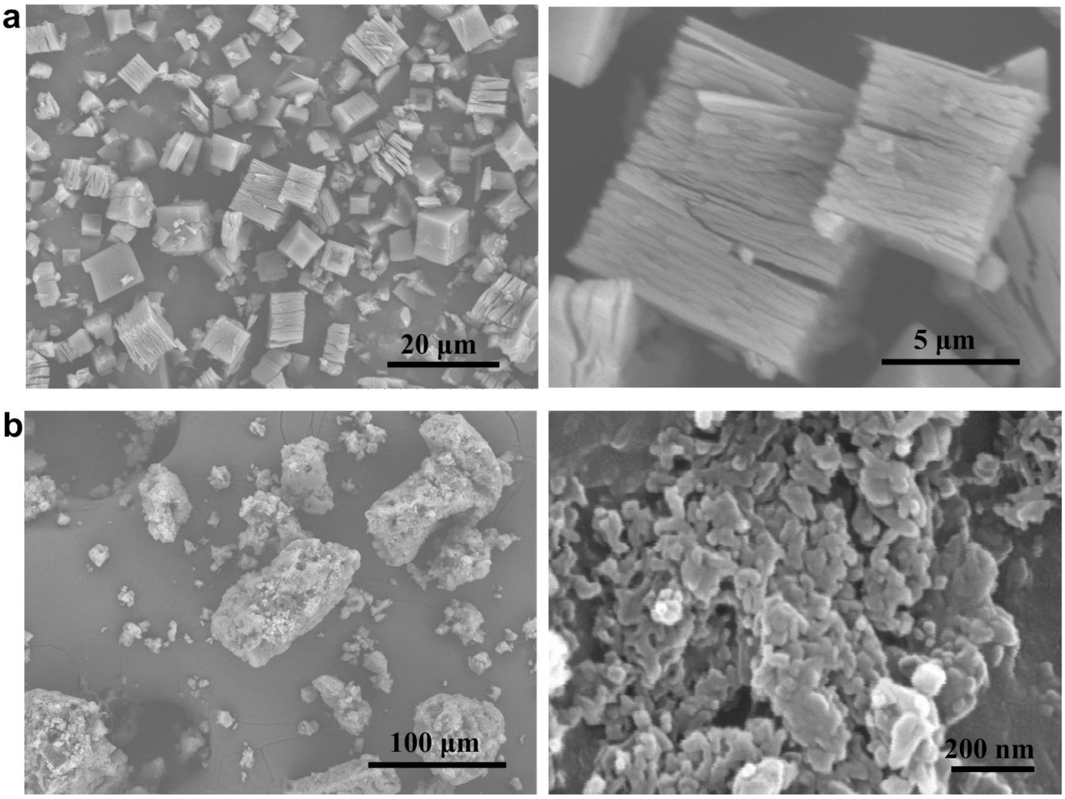

Supplementary Figure 9. SEM images of the original and transformed MOFs. a, CuBDC. **b**, Transformed MIL-53.

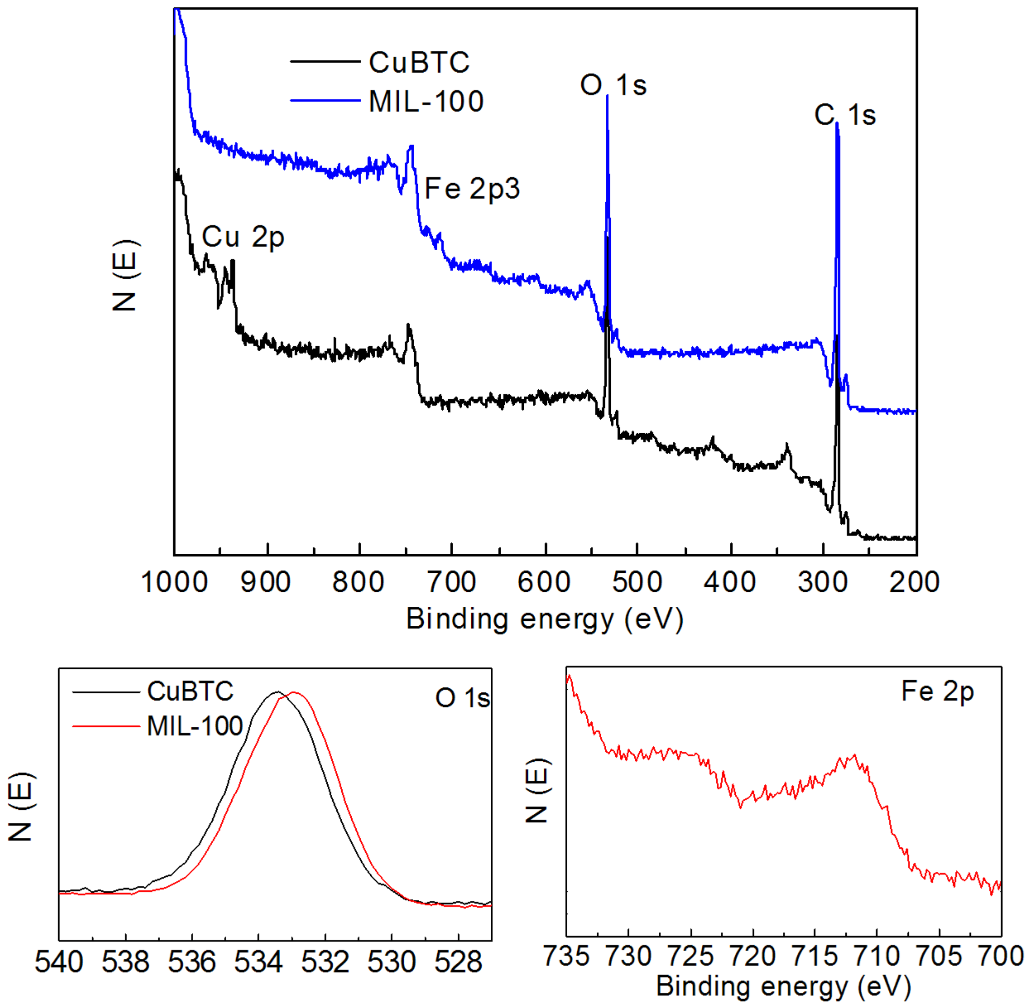

Supplementary Figure 10. XPS spectra of original CuBTC and MIL-100.

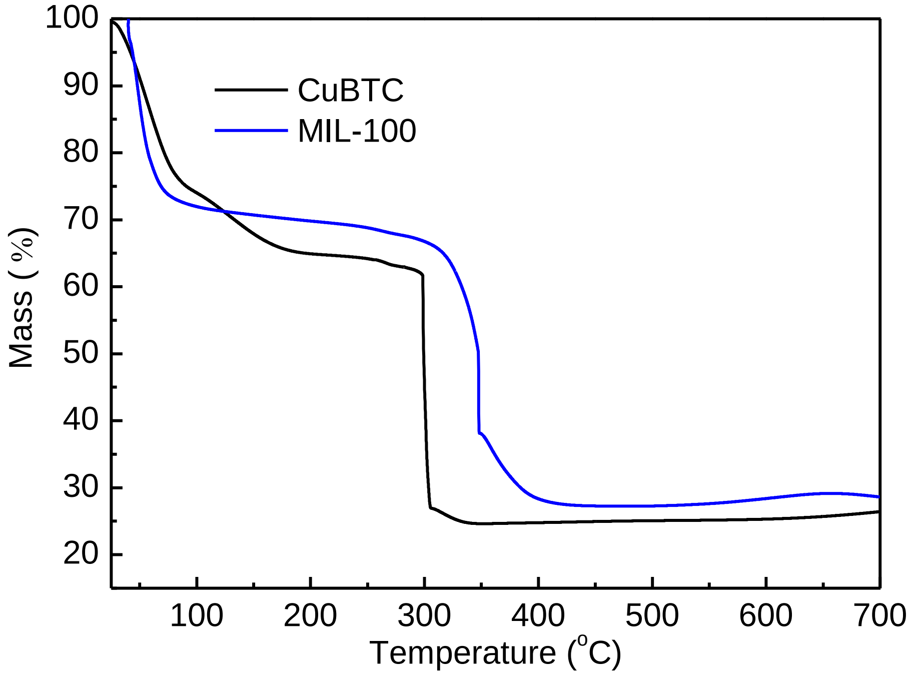

Supplementary Figure 11. TG curves of original CuBTC and MIL-100.

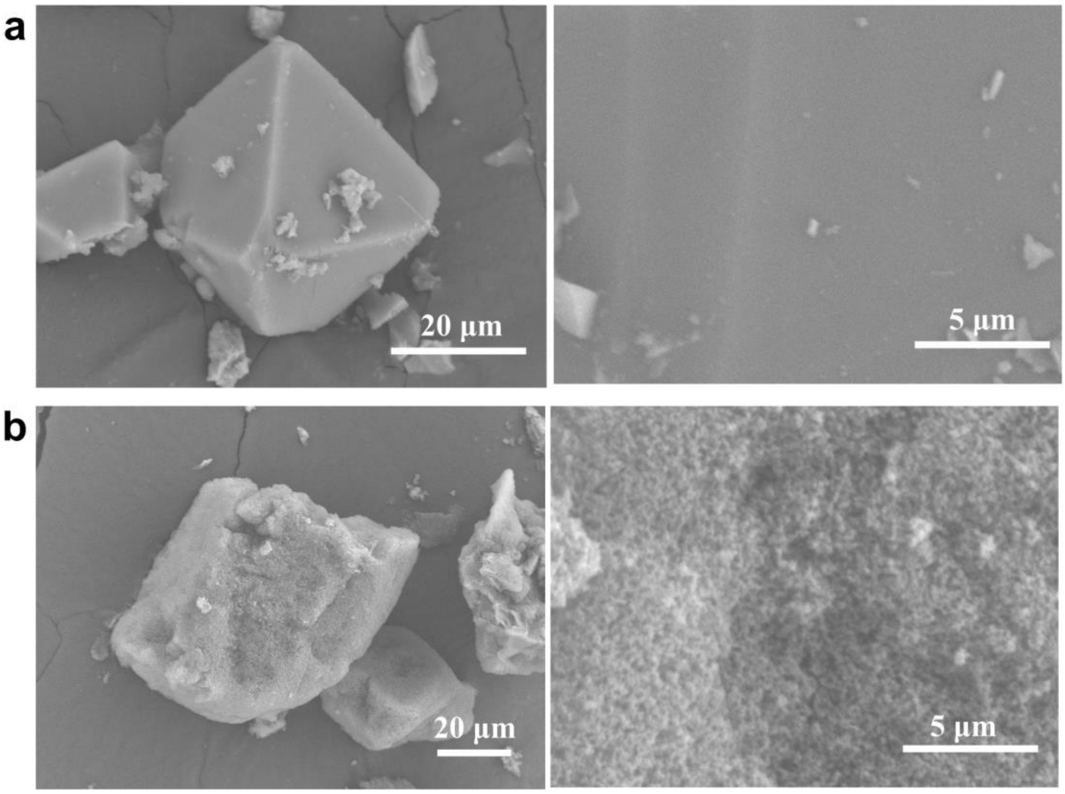

Supplementary Figure 12. SEM images of the CuBTC and MIL-100 after treated by ultrasound. a, CuBTC. **b**, MIL-100. After treated by ultrasond, MIL-100 still linked together tightly. This anti-ultrasonic property demonstrates that MIL-100 nanoparticles are grown together tightly, rather than simple accumulation.

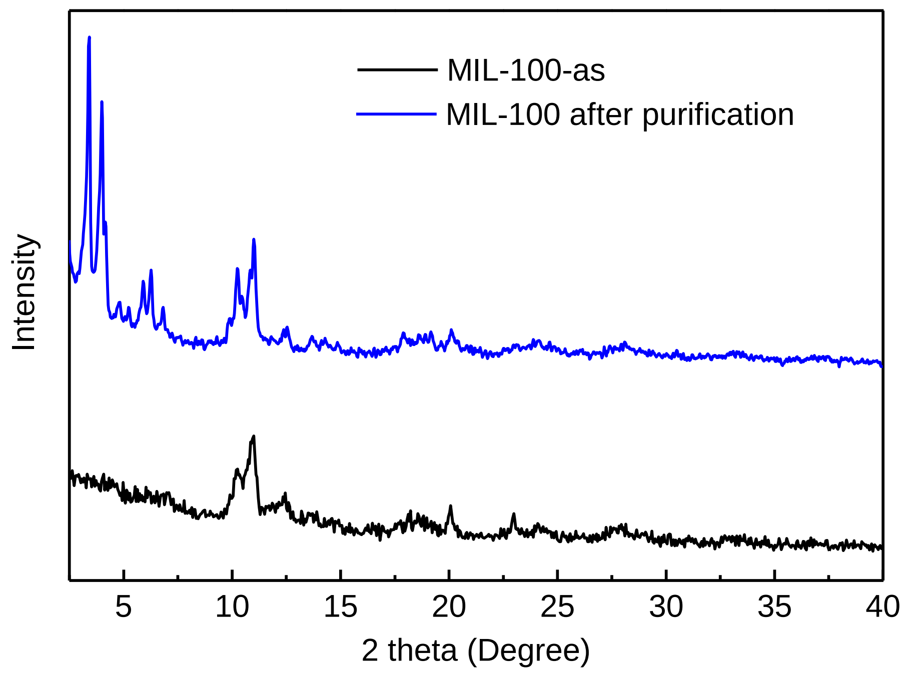

Supplementary Figure 13. XRD patterns of the as-synthesized MIL-100 and MIL-100 after purification. The relative intensities of XRD patterns of the as-synthesized MIL-100 shows a strong reduction compared with the MIL-100 after purification, especially at low angles, which may be attributed to the impregnation of amorphous $FeCl_3$. This phenomenon has also been observed for MIL-101 loaded with polyoxometalate[3,4].

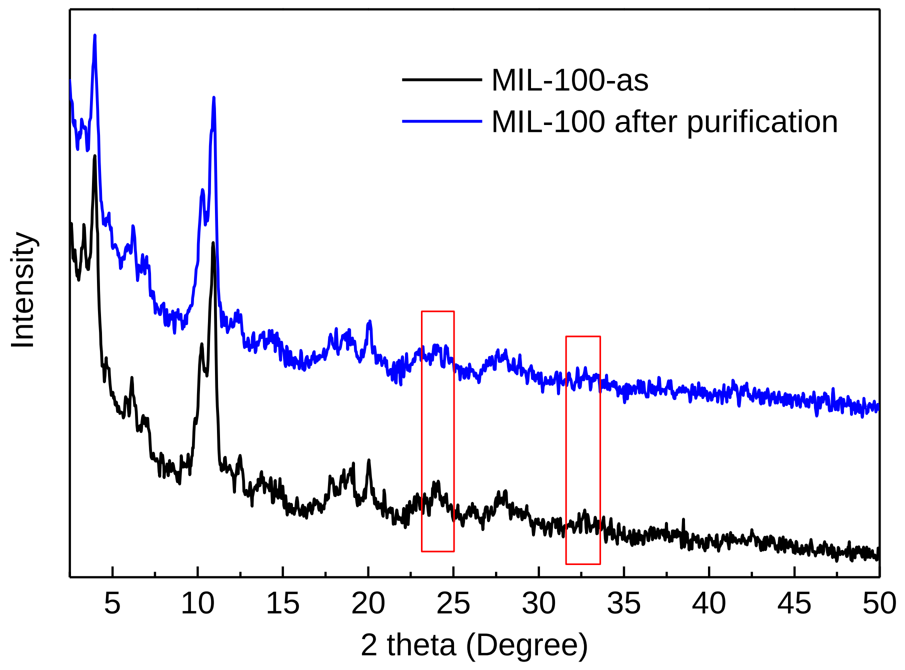

Supplementary Figure 14. XRD patterns of as-synthesized MIL-100 and MIL-100 after purification. The samples were calcined at 200 ℃ for 4 h. The new peaks in red boxes demonstrate the existence of the Fe_2O_3 in cavities of as-synthesized MIL-100.

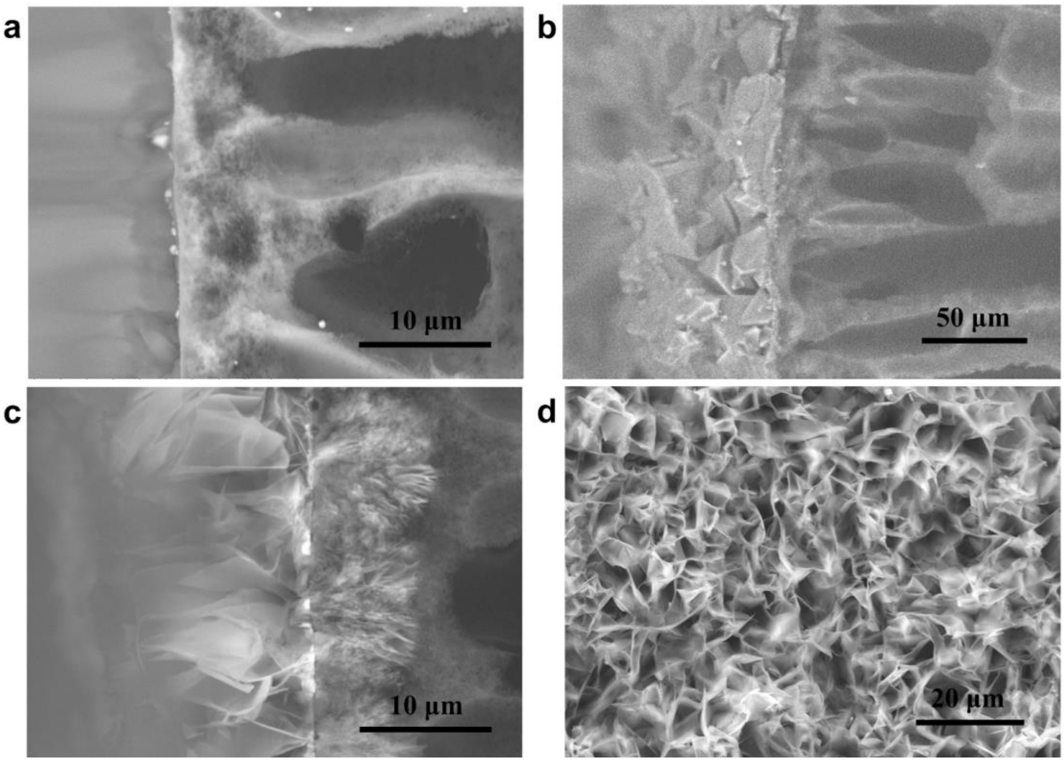

Supplementary Figure 15. SEM images of the substrate. a, PVDF hollow fiber. **b**, CuBTC membrane supported by dopamine modified hollow fiber; the CuBTC membrane is not continuous. **c**,**d**, Non-activation ZnO array on hollow fiber.

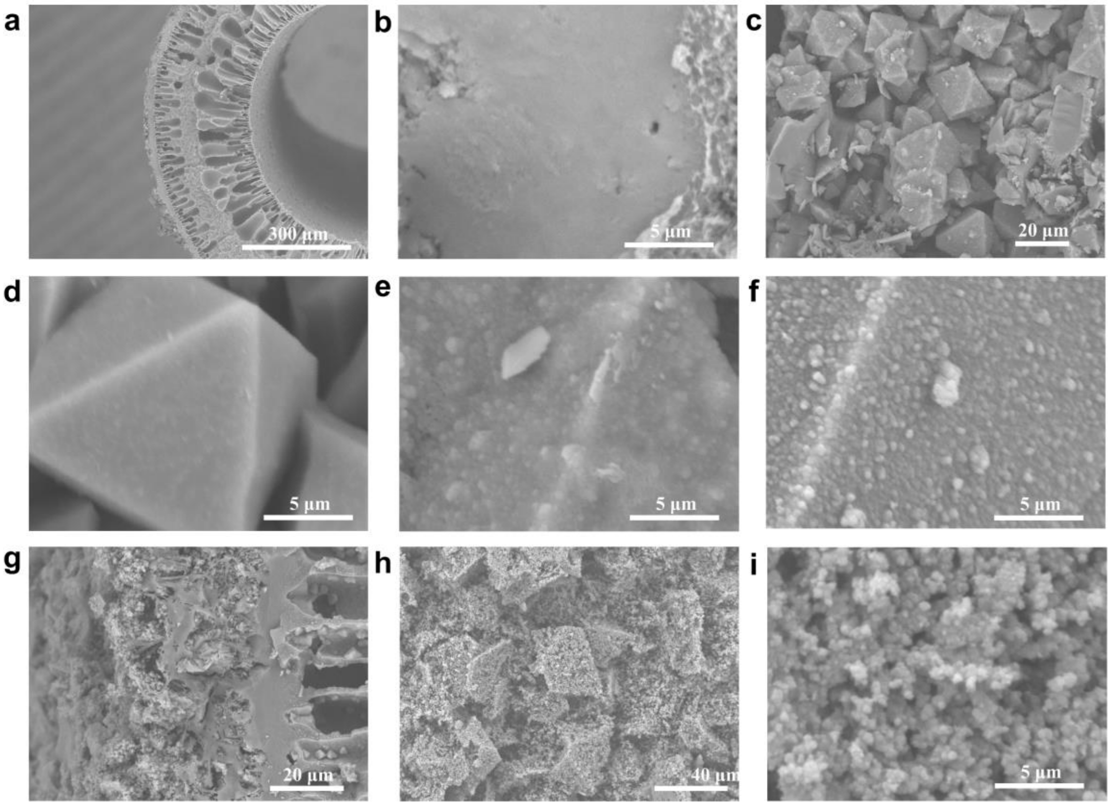

Supplementary Figure 16. SEM images of the membranes. **a**,**b**, Cross section and **c** top view SEM images of the dense transformed CuBTC/MIL-100 membrane. **e**,**d**,**f** Top view SEM images of original CuBTC membrane, transformed CuBTC/MIL-100 membrane, and transformed CuBTC/MIL-100 membrane after purification. Because the transformation was carried out under standing, the prepared membrane have not shown the superstructural composite surfaces and three-dimensional CuBTC shelf have been observed in particle transformation by shaking **g**, Cross section and **h**,**i** top view SEM images of CuBTC/MIL-100 membrane with transformation time of 48 h.

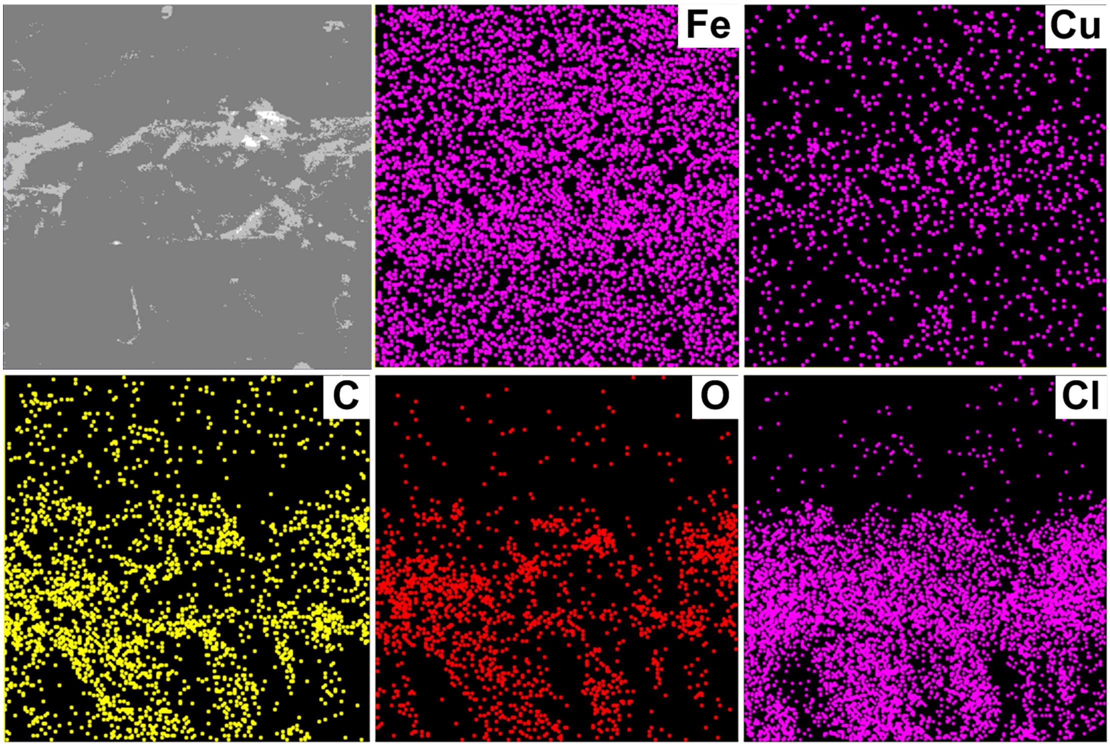

Supplementary Figure 17. EDS mapping. CuBTC/MIL-100 membrane after transformation for 12 h, Cu/Fe ratio is 24.5 %.

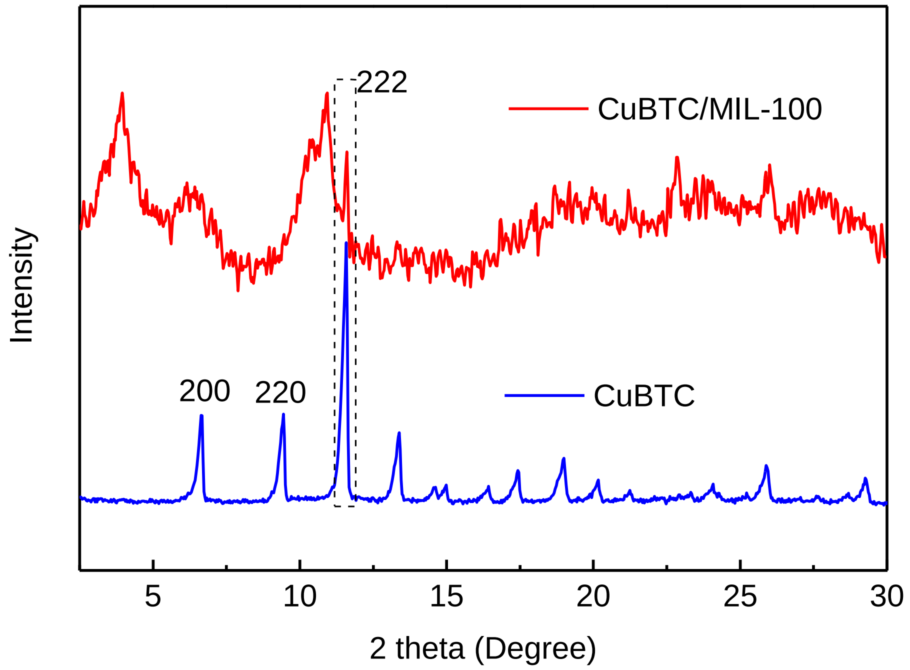

Supplementary Figure 18. XRD patterns of CuBTC membrane and CuBTC/MIL-100 membrane after transformation for 12 h. The peaks of the {222} facet of the CuBTC in CuBTC/MIL-100 demonstrate that the transformation is not complete.

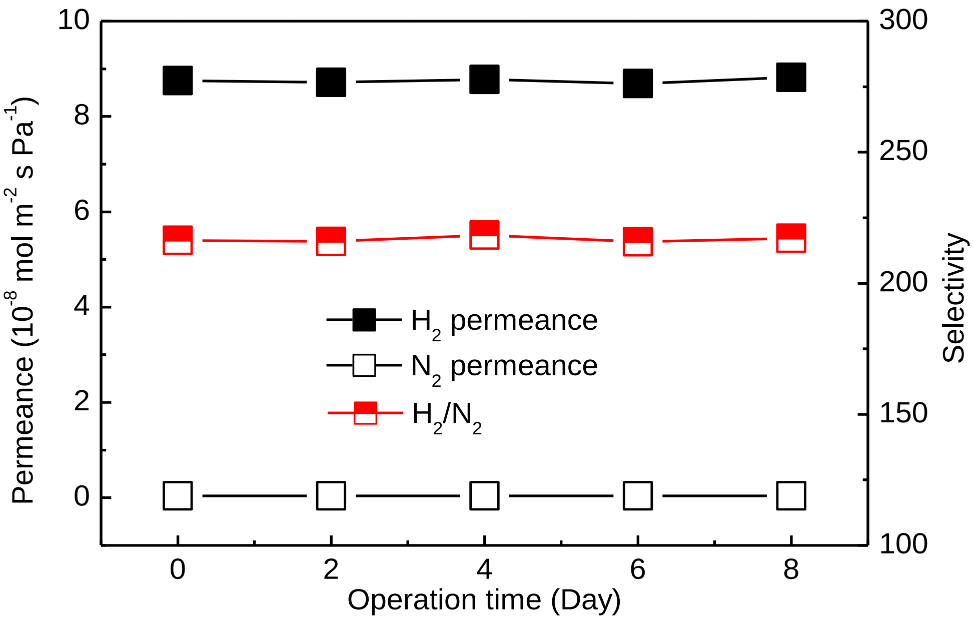

Supplementary Figure 19. H_2 and N_2 permeances and H_2/N_2 selectivity of CuBTC/MIL-100 membrane as a function of permeation time.

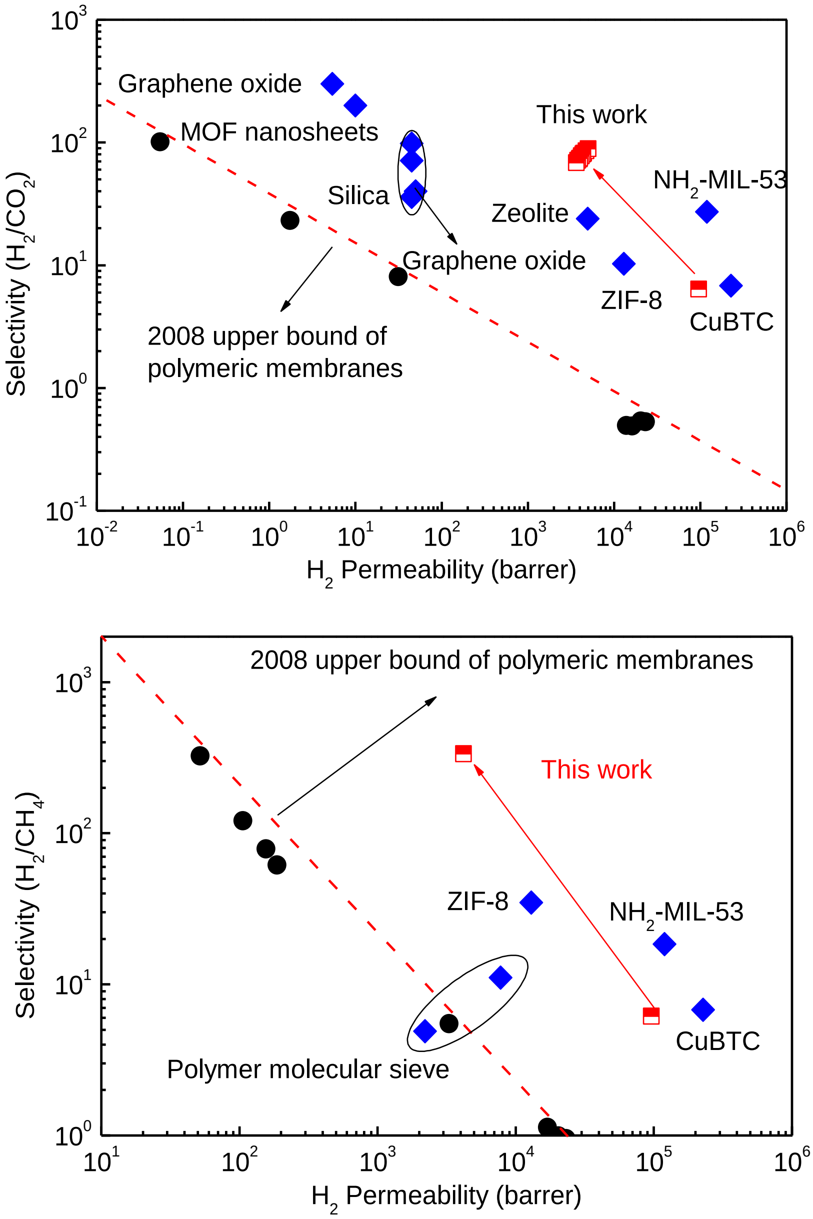

Supplementary Figure 20. Comparison of CuBTC/MIL-100 membranes with polymeric[5,6], silica[7], zeolite[8], other MOF[9,10,11], graphene oxide and $Zn_2(bim)_4$ nanosheet membranes[12-14] for H_2/CO_2 and H_2/CH_4 system. 1 barrer=3.348×10^{-16} mol m m^{-2} s^{-1} Pa^{-1}. The red dotted line is the Robeson's upper-bound reported in 2008[5].

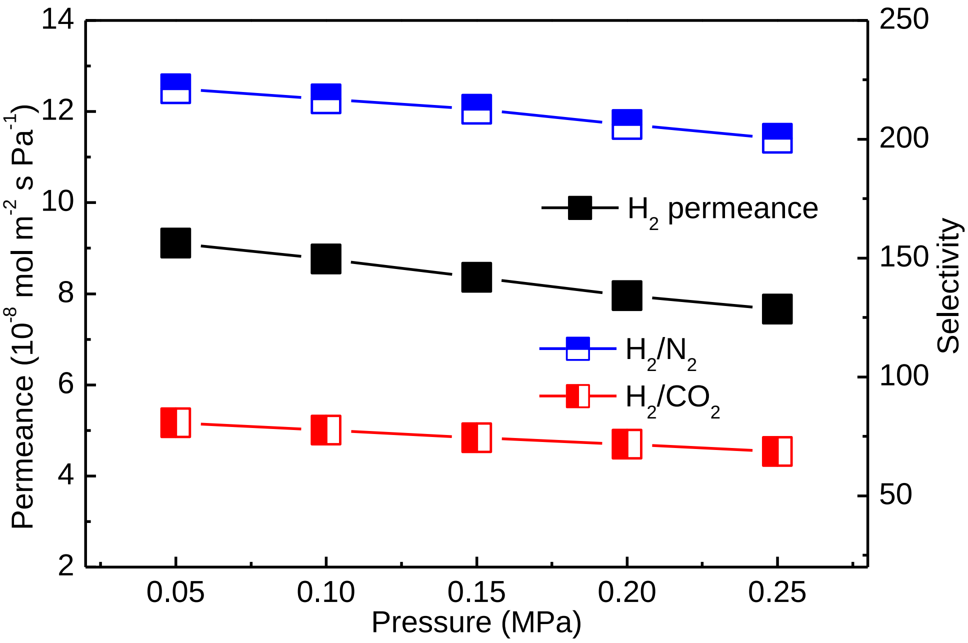

Supplementary Figure 21. Effect of feed pressure on permeances and H_2/CO_2 and H_2/N_2 selectivities of CuBTC/MIL-100 membrane.

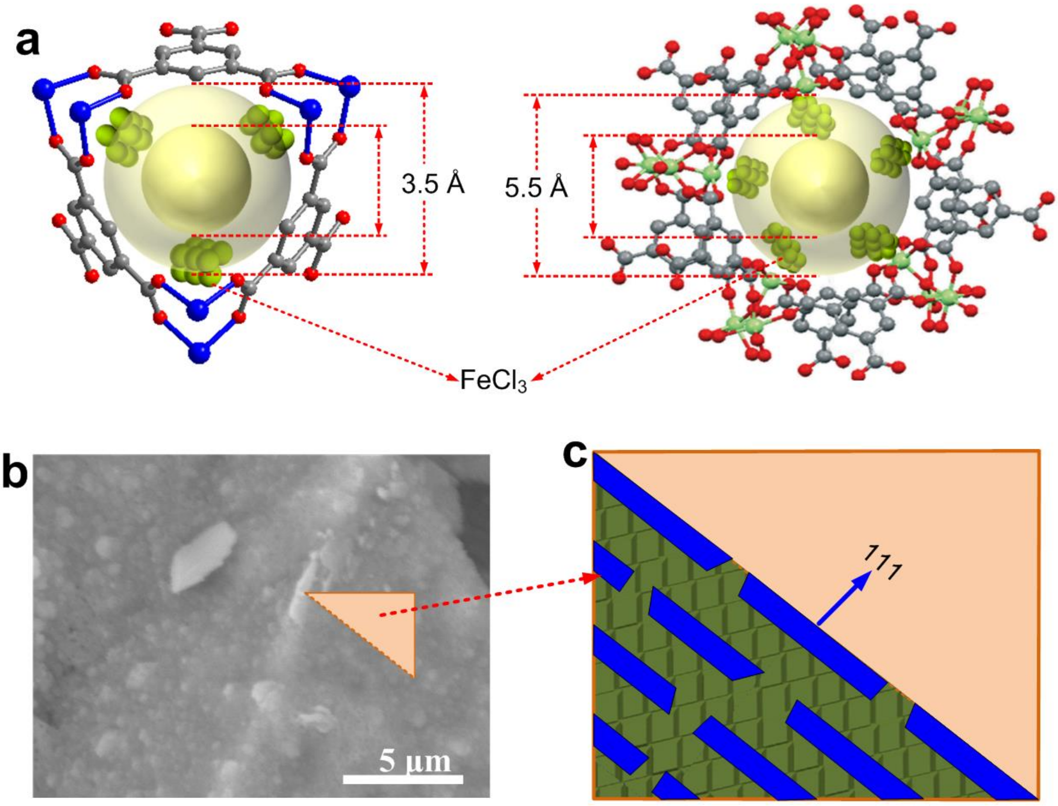

Supplementary Figure 22. The scheme diagrams of the dense CuBTC/MIL-100 membrane and their pore structures. The blue parts is the residual CuBTC with exposed {111} facets. The green part is the residual MIL-100. Because the reagents first enter into of the {100} facets of CuBTC, the triangular shaped windows with a diameter of 3.5 Å in the residual and exposed {111} facets is a main channel for gas to pass through. Moreover, the massive amorphous $FeCl_3$ in membrane can fill the gaps between the MIL-100 and CuBTC and occupy the cavities and pores of MOFs, and reduce the gas channel.

Supplementary Table 1. Synthetic conditions and the porosity of MIL-100 in reported literatures.

Methods	HF	Temp ℃	Time hour	S_{BET} $m^2\ g^{-1}$	$S_{Langmuir}$ $m^2\ g^{-1}$	V_{Total} cm^3/g	Ref
Hydrothermal	Yes	150	144		2800		15
Hydrothermal	Yes	160	8	2300		1.20	16
Hydrothermal	Yes	160	8	2047		0.83	17
Hydrothermal	Yes	150	144	1917		1.00	18
Microwave-hydrothermal	No	130	0.1	1350		0.86	19
Hydrothermal	Yes	160	8	2050		0.90	20
	No	160	12	1800		1.15	
Hydrothermal	No	130	72	1045			21
Hydrothermal	No	130	72		2800	0.88	4
Hydrothermal	No	150	24		2323	0.52	22
	Yes	150	24		2776	0.72	
Stirring-reflux	No	95	12	1836		1.16	23
Shaking	No	RT	12	1880		1.07	This work

Supplementary Table 2. Element content of the MIL-100 after purification or/and calcination. **A**, as-synthesized MIL-100. **B**, MIL-100 after purification in methanol for 5 cycles. **C**, as-synthesized MIL-100 after calcination at 200 ℃ for 4 hours. **D**, MIL-100 after purification in methanol for 5 cycles and calcination at 200 ℃ for 4 hours. **E**, MIL-100 after purification in methanol and hot water. **a**, EDS. **b**, Atomic absorption spectroscopy. The small amount of the Cu in MIL-100 demonstrate that the Cu enters into the solution during the transformation from CuBTC to MIL-100.

Element	A	B	C	D	E
C-K Atom %	46.21[a]	55.13	44.08	48.13	55.64
O-K Atom %	30.68	32.24	38.04	36.90	33.46
Cl-K Atom %	10.16	2.22	2.55	1.37	0.20
Fe-K Atom %	12.52 (98.38)[b]	10.11 (98.21)	14.82 (98.68)	13.30 (98.80)	10.66
Cu-K Atom %	0.43 (1.62)	0.29 (1.79)	0.51 (1.32)	0.30 (1.20)	0.04

Supplementary Table 3. Separation performance of the two additional membranes. **a**, The values were measured by constant-pressure method. **b**, The values in brackets were the H_2 permeance in H_2/CO_2 system and selectivities of binary mixtures and measured by Wicke-Kallenbach technique.

Membrane	H_2 permeance $\times10^{-8}$ mol m^{-2} s^{-1} Pa^{-1}	Selectivity		
		H_2/CO_2	H_2/N_2	H_2/CH_4
M2	9.5[a]	70.1	204.4	306.2
M3	7.9	85.7	230.2	354.8
	(7.4)[b]	(78.2)	(212.5)	(321.3)

Supplementary references

1. Irving, H. & Williams, R. J. P. The stability of transition-metal complexes. *J. Chem. Soc.* 3192-3210 (1953).
2. Carson, C. G. *et al*. Synthesis and structure characterization of copper terephthalate metal-organic frameworks. *Eur. J. Inorg. Chem.* 2338-2343 (2009).
3. Férey, G. *et al.* A chromium terephthalate-based solid with unusually large pore volumes and surface area. *Science* **309**, 2040-2042 (2005).
4. Canioni, R. *et al.* Stable polyoxometalate insertion within the mesoporous metal organic framework MIL-100(Fe). *J. Mater. Chem.* **21**, 1226-1233 (2011).
5. Robeson, L. M. The upper bound revisited. *J. Membr. Sci.* **320**, 390-400 (2008).
6. Carta, M. *et al.* An efficient polymer molecular sieve for membrane gas separations. *Science* **339**, 303-307 (2013).
7. de Vos, R. M. & Verweij, H. High-selectivity, high-flux silica membranes for gas separation. *Science* **279**, 1710-1711 (1998).
8. Guan, G., Tanaka, T., Kusakabe, K., Sotowa, K. & Morooka, S. Characterization of AIPO(4)-type molecular sieving membranes formed on a porous alpha-alumina tube. *J. Membr. Sci.* **214**, 191-198 (2003).
9. Guo, H. Zhu, G., Hewitt, I. J. & Qiu, S. “Twin copper source” growth of metal-organic framework membrane: $Cu_3(BTC)_2$ with high permeability and selectivity for recycling H_2. *J. Am. Chem. Soc.* **131**, 1646-1647 (2009).

10. Liu, Q., Wang, N., Caro, J. & Huang, A. Bio-inspired polydopamine: A versatile and powerful platform for covalent synthesis of molecular sieve membranes. *J. Am. Chem. Soc.* **135**, 17679-17682 (2013).

11. Zhang, F. *et al.* Hydrogen selective NH_2-MIL-53(Al) MOF membranes with high permeability. *Adv. Funct. Mater.* **22**, 3583-3590 (2012).

12. Peng, Y. *et al.* Metal-organic framework nanosheets as building blocks for molecular sieving membranes. *Science* **346**, 1356-1359 (2014).

13. Kim, H. W. *et al.* Selective gas transport through few-layered graphene and graphene oxide membranes. *Science* **342**, 91-95 (2013).

14. Li, H. *et al.* Ultrathin, molecular-sieving graphene oxide membranes for selective hydrogen separation. *Science* **342**, 95-98 (2013).

15. Horcajada, P. *et al.* Synthesis and catalytic properties of MIL-100(Fe), an iron(III) carboxylate with large pores. *Chem. Commun.* 2820-2822 (2007).

16. Yoon, J. W. *et al*. Controlled reducibility of a metal-organic framework with coordinatively unsaturated sites for preferential gas sorption. *Angew. Chem. Int. Ed.* **49**, 5949-5952 (2010).

17. Ahmed, I., Jun, J. W., Jung, B. K. & Jhung, S. H. Adsorptive denitrogenation of model fossil fuels with Lewis acid-loaded metal-organic frameworks (MOFs). *Chem. Eng. J.* **255**, 623-629 (2014).

18. Jeremias, F., Khutia, A., Henninger, S. K. & Janiak, C. MIL-100(Al, Fe) as water adsorbents for heat transformation purposes-a promising application. *J. Mater. Chem.* **22**, 10148-10151 (2012).

19. Márquez, A. G. . *et al.* Green microwave synthesis of MIL-100(Al, Cr, Fe) nanoparticles for thin-film elaboration. *Eur. J. Inorg. Chem.* 5165-5174 (2012).

20. Seo, Y. K. *et al*. Large scale fluorine-free synthesis of hierarchically porous iron(III) trimesate MIL-100(Fe) with a zeolite MTN topology. *Microporous Mesoporous Mater.* **157**, 137-145 (2012).

21. Wan, H. *et al*. Encapsulation of heteropolyanion-based ionic liquid within the metal-organic framework MIL-100(Fe) for biodiesel production. *ChemCatChem* **7**, 441-449 (2015).

22. Tan, F. *et al.* Facile synthesis of size-controlled MIL-100(Fe) with excellent adsorption capacity for methylene blue. *Chem. Eng. J.* **281**, 260-367 (2015).

23. Zhang, F. *et al.* Facile synthesis of MIL-100(Fe) under HF-free conditions and its application in the acetalization of aldehydes with diols. *Chem. Eng. J.* **259**, 183-190 (2015).
